# Topical Lidocaine During Airway Manipulation in Pediatric Anesthesia: A Systematic Review and Meta‐Analysis

**DOI:** 10.1111/pan.70077

**Published:** 2025-11-11

**Authors:** Elizabet Taylor Pimenta Weba, Gabriel Soares de Sousa, Alexandros Páris de Mesquita Ipácio, Christian Ken Fukunaga, Rafael Andrade Sampaio Silva, Marco Antonio Figueiredo Teixeira, Rafaela Machado Filardi, Carolina Magalhães Costa, Ricardo Vieira Carlos, Britta S. von Ungern‐Sternberg

**Affiliations:** ^1^ State University of Maranhão Tocantine Region Imperatriz Brazil; ^2^ Discipline of Anaesthesiology, Faculdade de Medicina Universidade de São Paulo São Paulo Brazil; ^3^ Instituto da Criança e do Adolescente, Hospital das Clínicas HCFMUSP, Faculdade de Medicina Universidade de São Paulo São Paulo Brazil; ^4^ Serviços Médicos de Anestesia, Hospital Sírio‐Libanês São Paulo Brazil; ^5^ FMABC University Center São Paulo Brazil; ^6^ Bahiana School of Medicine and Public Health Salvador Brazil; ^7^ Petrópolis Medical School Rio de Janeiro Brazil; ^8^ Neuromodulation Center and Center for Clinical Research Learning, Spaulding Rehabilitation Hospital and Massachusetts General Hospital, Harvard Medical School Boston Massachusetts USA; ^9^ Hepatology and Liver Transplantation, Hospital Sírio‐Libanês São Paulo Brazil; ^10^ Hepatology and Liver Transplantation, A. C. Camargo Cancer Center São Paulo Brazil; ^11^ Department of Anaesthesia and Pain Medicine Perth Children's Hospital Nedlands Western Australia Australia; ^12^ Institute for Paediatric Perioperative Excellence The University of Western Australia Perth Western Australia Australia; ^13^ Perioperative Care Program, Perioperative Medicine Team, Telethon Kids Institute Nedlands Western Australia Australia; ^14^ Division of Emergency Medicine, Anaesthesia and Pain Medicine The University of Western Australia Perth Western Australia Australia

**Keywords:** adverse events, airway management, lidocaine, pediatric anesthesia, topical drug administration

## Abstract

**Introduction:**

Lidocaine is widely used in pediatric anesthesia for airway topicalization to modulate undesirable airway and circulatory reflexes, yet its effectiveness remains unclear. Therefore, we aimed to perform a meta‐analysis evaluating the impact of topical lidocaine on respiratory adverse events in children undergoing airway management.

**Methods:**

PubMed, Embase, and Cochrane databases were systematically searched for studies comparing topical lidocaine with placebo, no intervention, or intravenous lidocaine for pediatric airway management. Statistical analysis was performed using R (version 4.4.1). Odds ratios (ORs) were used for binary outcomes and mean differences for continuous outcomes, with 95% confidence intervals (CIs) computed using a random‐effects model.

**Results:**

Fourteen randomized controlled trials comprising 1937 pediatric patients were included, of whom 917 (47%) received airway topicalization. In those receiving topical lidocaine, there was a significant reduction in the incidence of laryngospasm (OR 0.50; 95% CI 0.27 to 0.95; *p* = 0.033), desaturation (OR 0.49; 95% CI 0.25 to 0.98; *p* = 0.043), and sore throat (OR 0.31; 95% CI 0.16 to 0.58; *p* < 0.001). However, no significant differences were observed for bronchospasm (OR 0.50; 95% CI 0.11 to 2.35; *p* = 0.382), cough (OR 0.56; 95% CI 0.28 to 1.11; *p* = 0.099), severe cough (OR 1.30; 95% CI 0.18 to 9.51; *p* = 0.793), hoarseness (OR 1.41; 95% CI 0.17 to 11.96; *p* = 0.754), vomiting (OR 1.95; 95% CI 0.47 to 7.99; *p* = 0.355), and heart rate (beats/min) (MD 0.08; 95% CI −6.31 to 6.47; *p* = 0.98).

**Conclusion:**

Our findings suggest that topical lidocaine may reduce the incidence of undesirable airway reflexes such as laryngospasm, desaturation, and sore throat in children undergoing airway management. However, its benefit for other perioperative respiratory adverse events requires further investigation, especially in high‐risk populations.

**Trial Registration:**

PROSPERO registration number: CRD42024614863

**Abbreviations**
ASA: American Society of Anesthesiologists
CI: confidence interval(s)
ESAIC‐BJA: European Society of Anaesthesiology and Intensive Care and the British Journal of Anesthesia
GRADE: Grading of Recommendations Assessment, Development and Evaluation
MD: mean difference
OR: odds ratio
PRAEs: perioperative respiratory adverse events
PRISMA: Preferred Reporting Items for Systematic Reviews and Meta‐Analyses
PROSPERO: International Prospective Register of Systematic Reviews
RCT: randomized controlled trial(s)
RoB‐2: Cochrane Collaboration Risk of Bias 2 Tool
ROBINS‐I: Risk of Bias in Non‐randomized Studies of Interventions tool
SD: standard deviation(s)
URTI: upper respiratory tract infection

## 1 Introduction

Lidocaine is widely used in pediatric anesthesia for airway topicalization, a technique that involves applying the anesthetic to the respiratory mucosa to facilitate medical procedures such as intubation and bronchoscopy [1, 2]. While airway manipulation is often unavoidable during these interventions and may lead to protective physiological responses (e.g., coughing, laryngospasm), lidocaine may be an effective and straightforward strategy for counteracting these negative effects in children [3, 4].

In this context, applying topical lidocaine using various administration techniques aims to modulate undesirable airway and circulatory reflexes during the airway procedure and throughout the recovery period [5]. However, despite its common use, the effectiveness of lidocaine for airway topicalization remains controversial. Some studies suggest that it can significantly reduce the incidence of perioperative respiratory adverse events (PRAEs) in children undergoing elective airway procedures [6, 7, 8]. In contrast, other research indicates that topical anesthesia may offer no benefits and even reports an increased incidence of perioperative airway complications due to irritation caused by the spray itself when used as an adjunct to routine endotracheal intubations [9].

Since the publication of prior meta‐analyses evaluating the role of topical airway lidocaine in patient comfort and its safety during procedures, subsequent studies have been published, including randomized clinical trials (RCTs) [10, 11]. Additionally, previous meta‐analyses have not specifically investigated the incidence of perioperative adverse effects in children based on different application methods and types of airway intervention. In light of this controversy, we performed an updated meta‐analysis to evaluate the efficacy of airway topicalization with lidocaine compared with placebo, no intervention, and intravenous lidocaine in preventing PRAEs in the pediatric population during airway management.

## 2 Methods

### 2.1 Study Protocol

This systematic review and meta‐analysis was conducted in accordance with the Preferred Reporting Items for Systematic Reviews and Meta‐Analyses (PRISMA) Statement guidelines and the Cochrane Collaboration Handbook for Systematic Reviews of Interventions [12, 13]. The protocol for this study was registered in the International Prospective Register of Systematic Reviews (PROSPERO) in November 2024, under registration number CRD42024614863.

### 2.2 Eligibility Criteria

Inclusion of studies was guided by the following eligibility criteria: (1) full‐text published articles; (2) RCTs or non‐randomized cohorts comparing topical lidocaine with placebo, no intervention, or intravenous lidocaine anesthesia; (3) patients under 18 years old undergoing airway manipulation; and (4) reporting any outcomes of interest. Authors' classifications within the included studies were used to define the age ranges for the pediatric population. Exclusion criteria were: (1) non‐original articles (i.e., letters, editorials, comments, reviews, and meta‐analyses); (2) conference abstracts; (3) studies including patients not undergoing airway management; and (4) studies reporting no outcomes of interest. For the main quantitative synthesis, only RCTs were included due to their higher methodological quality and lower risk of bias. Observational studies addressing the same research question were identified and included only as Supporting Information for exploratory and contextual analyses.

### 2.3 Search Strategy and Data Extraction

A systematic search of the medical literature was conducted across three databases: PubMed, Embase, and Cochrane Library. Studies meeting eligibility criteria and published from inception to October 2024 were evaluated. The complete and adapted search strategy for each database is detailed in the Supporting Information. The references from all included studies, previous systematic reviews, and meta‐analyses were also searched manually for any additional studies meeting eligibility criteria.

Two authors (E.T.; A.P.) independently extracted variables of interest from the eligible studies using a standardized Microsoft Excel sheet and reviewed the full publications, including all Supporting Information. Data extraction included the number and characteristics of participants in the intervention and control groups, type of topical lidocaine formulation, control description, type of airway management, surgery/procedure characteristics, patients' American Society of Anesthesiologists (ASA) physical status classification, reported outcomes, and the respective means and standard deviations (SDs) or frequencies. Any discrepancies were resolved through consensus among these authors or through deliberation with senior team members (R.M.; G.S.). Outcome data were double‐checked, consolidated, and included in the meta‐analysis software. For eligible non‐randomized cohort studies, data were collected separately and presented in the Supporting Information to provide additional context, without inclusion in the primary meta‐analysis.

The trial by Schebesta et al. was organized into two groups to enhance the analysis: one group included children experiencing ongoing or recent upper respiratory tract infections (URTI), while the other comprised children without such infections [7]. Moreover, the trial conducted by O'Neil et al. reported two groups regarding the incidence of cough: one group comprised patients receiving adjuvant morphine, while the other included patients who did not receive any antitussive agent with topical lidocaine. To minimize confounding factors, only data pertaining to the non‐morphine group were extracted and included in the statistical analysis [14].

### 2.4 Endpoints and Subgroup Analysis

The primary endpoints of this meta‐analysis were (1) laryngospasm, (2) cough and (3) desaturation. We also evaluated the following secondary outcomes: (4) bronchospasm, (5) severe cough, (6) hoarseness, (7) vomiting, and (8) heart rate (beats/min). We utilized each author's definitions to identify events of desaturation and laryngospasm despite the variability in these criteria across different trials. Additionally, the documentation of respiratory adverse event occurrences was assessed using the variable time intervals established by the studies. Observational and randomized data were integrated only for the analysis of primary outcomes and are presented exclusively in the Supporting Information.

All prespecified subgroup analyses were performed exclusively using data from RCTs. These analyses included stratification according to the formulation of topical lidocaine, which consisted of: (1) spray, (2) gel or cream, (3) local injectable solution, and (4) nebulization. Studies were also subdivided based on the presence or absence of respiratory tract infections, as well as the concentration of lidocaine used (1% or 2%). Age was another factor considered, with participants classified as either preschoolers or school‐aged children. Lastly, the studies were grouped by type of airway manipulation, which included endotracheal intubation/extubation, laryngeal mask airway, and bronchoscopy.

### 2.5 Risk of Bias Assessment

The methodological quality of the included studies was assessed using the Cochrane Collaboration Risk of Bias 2 (RoB‐2) tool for RCTs [15]. For non‐randomized studies, the Risk of Bias in Non‐randomized Studies of Interventions (ROBINS‐I) tool was employed [16]. The domains assessed in the RoB‐2 tool were bias (1) from the randomization process, (2) due to deviations from intended interventions, (3) due to missing outcome data, (4) in the measurement of the outcomes and (5) in the selection of the reported result. In contrast, the domains evaluated in the ROBINS‐I were biased (1) due to confounding, (2) in the selection of participants, (3) in the classification of interventions, (4) deviations from intended interventions, (5) due to missing data, (6) in the measurement of outcomes and (7) in the selection of the reported result.

Based on assessments, the RoB‐2 tool classified studies into three categories according to their risk of bias and score: high risk, some concerns, or low risk of bias. Similarly, the ROBINS‐I tool categorized studies as having serious risk, moderate risk, or low risk of bias. The evaluations were independently carried out by two authors (C.K.; M.A.), with disagreements resolved through consensus after discussing reasons for the discrepancy.

### 2.6 Statistical Analysis

Endpoints were analyzed using odds ratios (ORs) for categorical endpoints and mean differences (MDs) for continuous outcomes with 95% confidence intervals (CIs) to compare treatment effects. We assessed heterogeneity using the Cochrane Q statistic, and Higgins and Thompson's *I* ^2^ using a restricted maximum‐likelihood estimator; *p* < 0.10 and *I* ^2^ > 25% were considered significant for heterogeneity. Der Simonian and Laird random‐effects models were used based on the assumption of varying effect sizes in the selected population [17]. The reported *p*‐values are two‐sided, and we made no adjustments for multiple testing. The guidelines from the Cochrane Handbook for Systematic Reviews of Interventions were used for data manipulation and conversion [13]. We conducted leave‐one‐out sensitivity analyses using only RCTs for the primary outcomes to assess the effects of influential studies on the pooled analyses. Additionally, meta‐regression analyses were performed exclusively with RCT data to explore the effects of potential moderators on the outcome variable. Statistical analyses were performed using R statistical software, version 4.4.0 (R Foundation for Statistical Computing). We used the packages readr, readxl, dplyr, and tidyverse for data management [18, 19, 20, 21]; meta, metafor, and metadat for meta‐analysis [22, 23, 24], and ggplot2, forestplot, and gridBase for data visualization [25, 26, 27]. The quality of evidence for the primary outcomes was assessed according to the Grading of Recommendation, Assessment, Development and Evaluations (GRADE) guidelines. RCTs were categorized into four levels of evidence quality: high, moderate, low, and very low [28, 29].

## 3 Results

### 3.1 Study Selection and Characteristics

**FIGURE 1:**
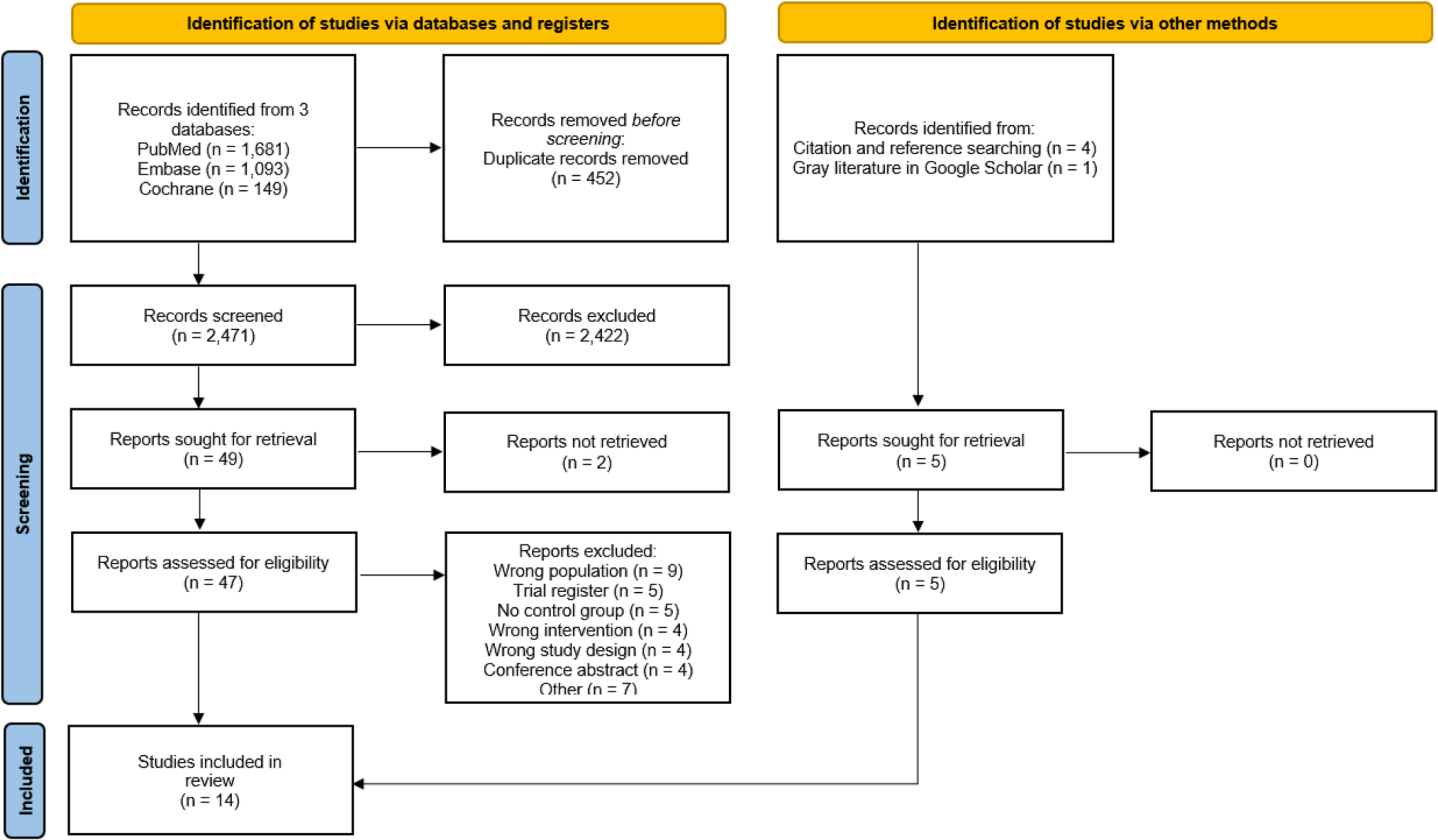
PRISMA flow diagram of study screening and selection.

As exhibited in Figure 1, the initial screening yielded a total of 2923 records. After removing duplicates and ineligible studies by title or abstract, 47 studies were selected for full‐text review. From these fully reviewed articles, nine studies were included in this meta‐analysis [6, 7, 8, 9, 14, 30, 31, 32, 33]. Additionally, using a background snowballing method, four further studies were identified [34, 35, 36, 37], along with one study sourced from gray literature via Google Scholar [36]. Ultimately, a total of 14 RCTs were included in this meta‐analysis [6, 7, 8, 9, 14, 30, 31, 32, 33, 34, 35, 36, 37, 38], comprising data from 1937 pediatric patients. In addition, three non‐randomized cohort studies (3882 children) were identified and analyzed in combination with the RCTs exclusively in the Supporting Information, without inclusion in the main quantitative synthesis [39, 40, 41].

Baseline characteristics of the RCTs are summarized in Table 1, while those of the non‐randomized studies are shown in Table S1. Amongst the RCTs, a total of 917 (47%) patients were assigned to the intervention group and 1020 (53%) to the control group. The population consisted of 923 (47%) males and had a mean (SD) age of 5.27 (2.81) years. Significant variability was observed across studies regarding the procedures performed, types of control groups, concentrations and formulations of topical lidocaine, and airway management methods. Specifically, 1109 (61%) patients underwent tracheal intubation or extubation, 658 (34%) received a laryngeal mask, and 170 (5%) underwent flexible bronchoscopy.

**TABLE 1:** Baseline characteristics of included studies.

Study, year	Design	Patients, *n*	Male^a^, %	Age^a^, years	Weight, kg	TAL concentration and formulation	Control description	Airway management	Surgery/Procedure characteristics	ASA classification, *n*
		TAL/CTRL	TAL/CTRL	TAL/CTRL	TAL/CTRL					TAL/CTRL
Behzadi [9], 2010	RCT	88/88	52.3/40.9	7 (5–10)/7 (5–10)	NR	2% Intracuff lidocaine injection	IV lidocaine + intracuff saline in ETT	ETI	Adenotonsillectomy	ASA I–II: 176
Gharaei [33], 2015	RCT	60/64	48.3/57.8	3.8 ± 1.2/3.8 ± 1.3	17.4 ± 3.2/16.3 ± 3.6	2% Lidocaine gel	IV lidocaine	LMA	Full ophthalmic examination	NR
Jarraya [38], 2024	RCT	60/60	85/78.3	3.28 ± 1.43/3.38 ± 1.38	15.13 ± 5.8/15.32 ± 5.2	2% Nebulized lidocaine	Nebulized saline	FB	Pediatric ambulatory ilioinguinal surgery	ASA I: 58 ASA II: 2/ASA I: 58 ASA II: 2
Koç [36], 1998	RCT	33/101	45.4/CTRL 1: 50.7 CTRL 2: 50 CTRL 3: 44.1	TAL: 7.1 ± 1.7/CTRL1: 6.7 ± 1.3 CTRL 2: 6.3 ± 0.8 CTRL 3: 7.6 ± 2.4	NR	2% Lidocaine spray	CTRL 1: Normal saline solution used topically CTRL 2: Normal saline solution used IV CTRL 3: IV lidocaine	ETI/EET	Tonsillectomy and/or Adenoidectomy	NR
Li [8], 2016	RCT	162/160	57.4/55.6	4.9 ± 3.4/5.1 ± 3.6	18.9 ± 5.9/21.3 ± 6.6	2% Lidocaine spray	Sterile saline spray	ETI	Elective surgical procedures under general anesthesia	ASA I: 98 ASA II: 49 ASA III: 15/ASA I: 95 ASA II: 52 ASA III: 13
Mussavi [32], 2015	RCT	30/30	56.6/46.6	35.3 ± 25.7 h/32.9 ± 24.3 h	2.11 ± 1.077/1.915 ± 0.854	6.5% Lidocaine spray	Saline spray	ETI	Neonates with GA > 30 weeks and without significant comorbidities	NR
O'Neill [14], 1994	RCT	63/57	NR	4.6 ± 3.8/4.0 ± 3.5	19.1 ± 11.2/17.8 ± 10.7	2% Viscous lidocaine solution	K‐Y Jelly	LMA	LMA suitable minor surgery	NR
Oliveira [31], 2024	RCT	50/50	66/56	7.2 ± 2.4/7.1 ± 2.6	29.8 ± 12.7/31.1 ± 14.7	1% Intracuff lidocaine solution + IV saline	Air‐filled cuff in ETT + IV saline	ETI	Tonsillectomy or Adenotonsillectomy	ASA‐I: 30 ASA‐II: 10/ASA I: 33 ASA II: 7
Peñaloza [35], 1998^b^	RCT	10/10	60	3.5 (1–8)	15.46	NR% Nebulized lidocaine	Placebo drug	ETI	Surgical procedures under general anesthesia	ASA I: 17 ASA II: 13
Schebesta [7], 2010	RCT	12/20	NR	5.2 ± 2.0/4.1 ± 2.6	16.4 ± 6.3/16.2 ± 5.9	2% Lidocaine gel	Placebo gel	LMA	Routine surgical procedures	ASA I: 12/ASA I: 20 ASA II: 21
Shafa [30], 2019	RCT	25/25	72/72	2.52 ± 1.87/2.60 ± 1.77	NR	1% Nebulized lidocaine	Nebulized saline	FOB	Diagnostic therapeutic procedures	ASA I and ASA II
Soares [34], 2017	RCT	82/82	59.7/56	0.5% TAL group: 7 [3–13] 1% TAL group: 8 [3–13]/CTRL 1: 7 [3–13] CTRL 2: 7 [3–13]	0.5% TAL group: 25 [13–70] 1% TAL group: 24 [12–75]/CTRL 1: 20 [11–78] CTRL 2: 25 [13–64]	0.5% and 1% Intracuff alkalinized lidocaine solution	CTRL 1: Air‐filled cuff CTRL 2: Saline‐filled cuff	EET	Elective surgical procedures under general anesthesia	ASA‐I: 58 ASA‐II: 24/ASA I: 53 ASA II: 29
Staffel [35], 1991	RCT	66/67	NR	10	NR	4% Lidocaine solution	No TAL	ETI	Adenoidectomy, tonsillectomy or adenotonsillectomy	NR
Sun—LA [6], 2021	RCT	96/95	47.9/51.6	3.9 ± 1.1/3.9 ± 1.2	16.6 ± 3.0/16.1 ± 2.7	NR% Lidocaine cream applied to cuff	Hydrosoluble lubricant applied to cuff	LMA	Squint correction	ASA I: 96/ASA I: 95
Sun—LD [6], 2021	RCT	97/94	47.4/51.5	3.9 ± 1.1/3.8 ± 1.2	16.4 ± 2.8/16.0 ± 2.8	NR% Lidocaine cream applied to cuff	Hydrosoluble lubricant applied to cuff	LMA	Squint correction	ASA I: 97/ASA I: 94

Abbreviations: ASA, American Society of Anesthesiologists; CTRL, control; EET, endotracheal extubation; ETI, endotracheal intubation; ETT, endotracheal tube; FB, flexible bronchoscopy; FOB, fiber optic bronchoscopy; GA, gestational age; IV, intravenous; LA, lidocaine cream smeared to the cuff of the LMA before insertion, with mask removal in the awake state; LD, lidocaine application and LMA removal under deep anesthesia; LMA, laryngeal mask airway; NMB, neuromuscular blockade; NR, not reported; RCT, randomized controlled trial; TAL, topical lidocaine.

^a^ Mean ± standard deviation or mean (range) or median [range].

^b^ Overall trial baseline characteristics were based on the total study population.

### 3.2 Pooled Analysis of All Outcomes

#### 3.2.1 Major Perioperative Respiratory Adverse Events

**FIGURE 2:**
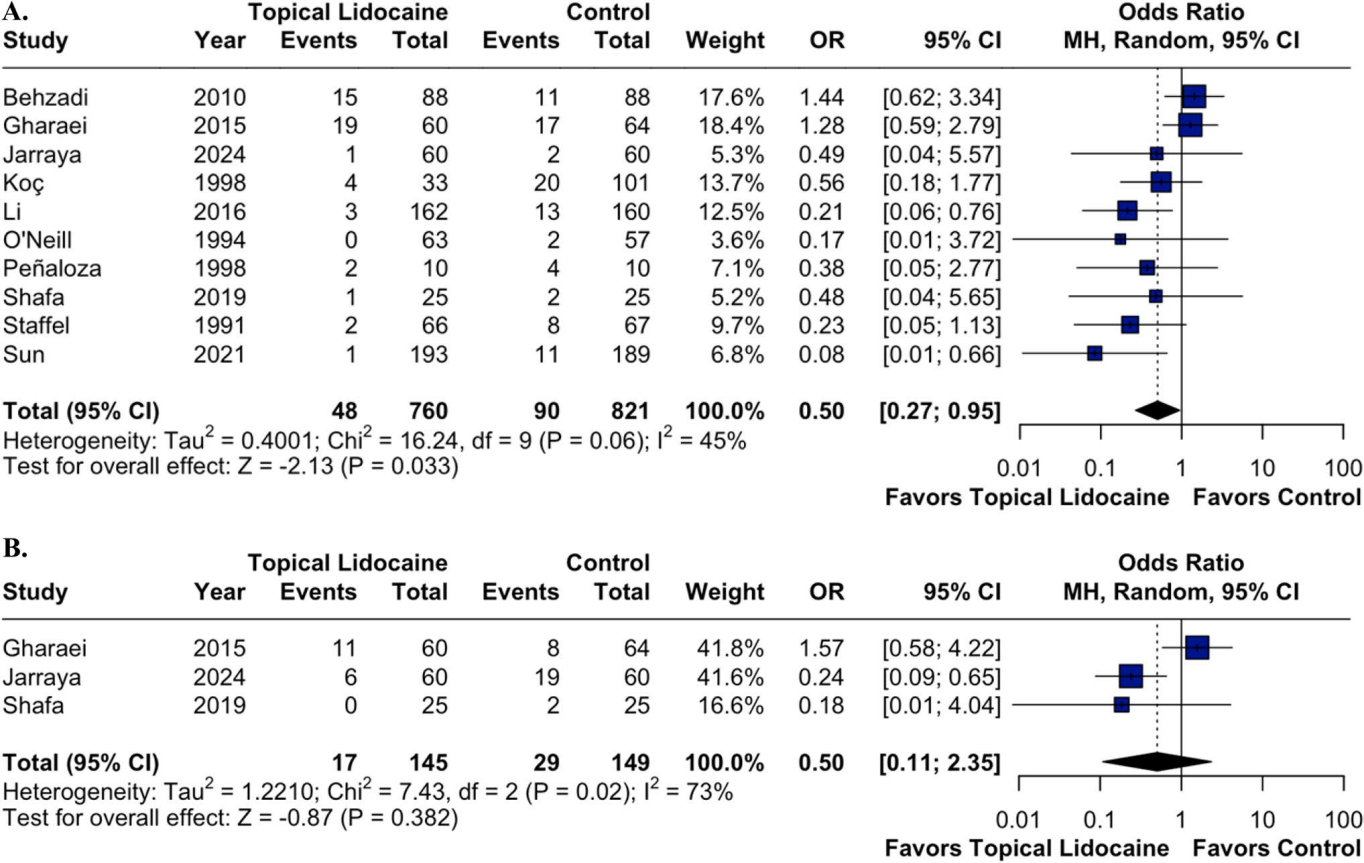
Topical lidocaine was associated with a significantly lower incidence of laryngospasm (A), but there was no significant reduction in the occurrence of bronchospasm (B) compared with the control group.

The incidence of laryngospasm was assessed in 10 RCTs involving 1581 patients. In those receiving topical lidocaine, there was a significantly lower incidence of laryngospasm compared with the control group (OR 0.50; 95% CI 0.27 to 0.95; *p* = 0.033; *I* ^2^ = 45%; Figure 2A). In contrast, there was no statistically significant difference in the incidence of bronchospasm between the groups (OR 0.50; 95% CI 0.11 to 2.35; *p* = 0.382; *I* ^2^ = 73%; Figure 2B).

#### 3.2.2 Coughing Phenomena

**FIGURE 3:**
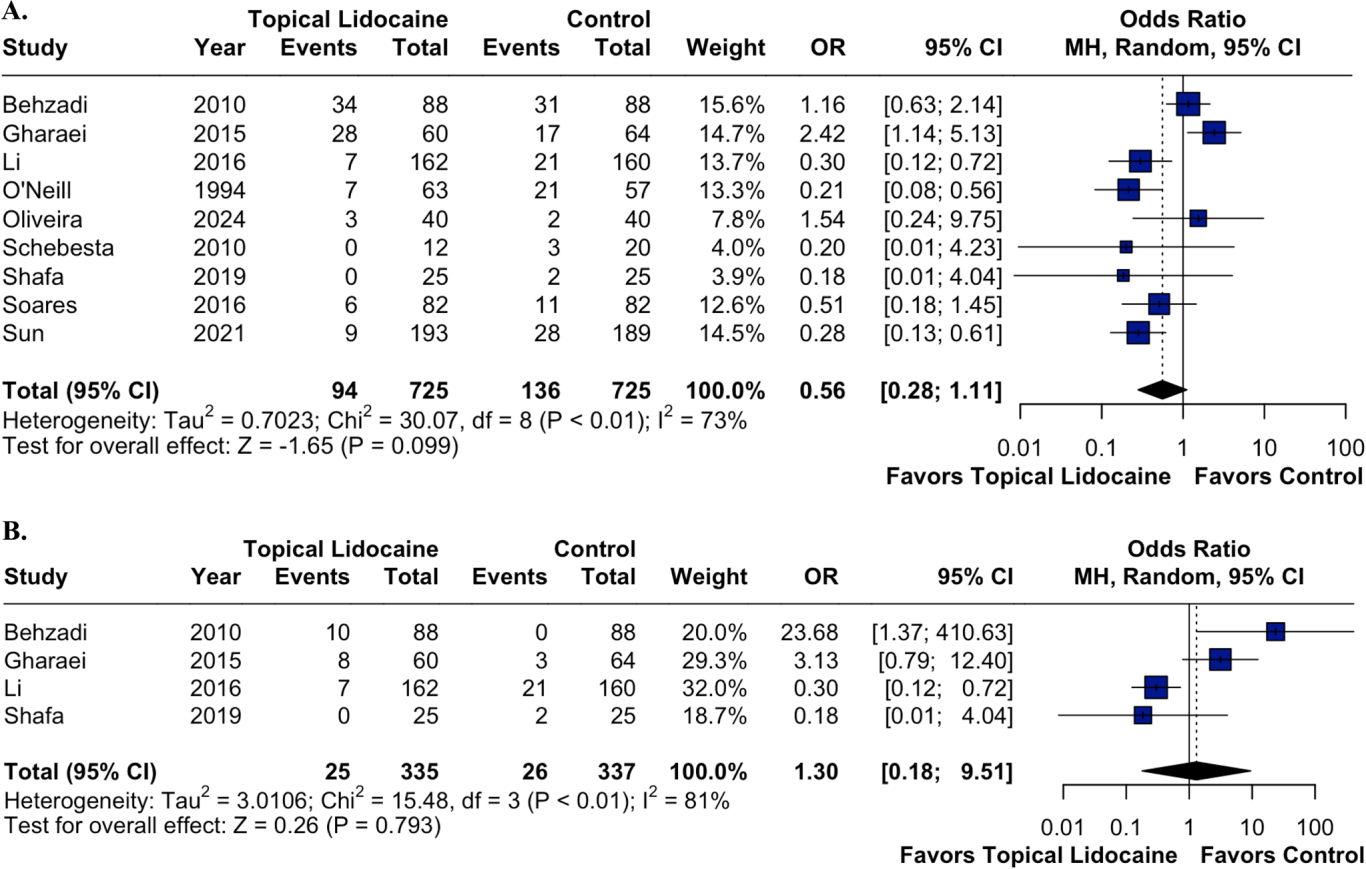
The composite of cough (A) and severe cough (B) was not significantly different between the groups.

Coughing was reported in nine RCTs including 1450 children. There was no statistically significant difference between the topical lidocaine and control groups regarding the occurrence of cough (OR 0.56; 95% CI 0.28 to 1.11; *p* = 0.099; *I* ^2^ = 73%; Figure 3A) and severe cough (OR 1.30; 95% CI 0.18 to 9.51; *p* = 0.793; *I* ^2^ = 81%; Figure 3B).

#### 3.2.3 Sore Throat Events

**FIGURE 4:**
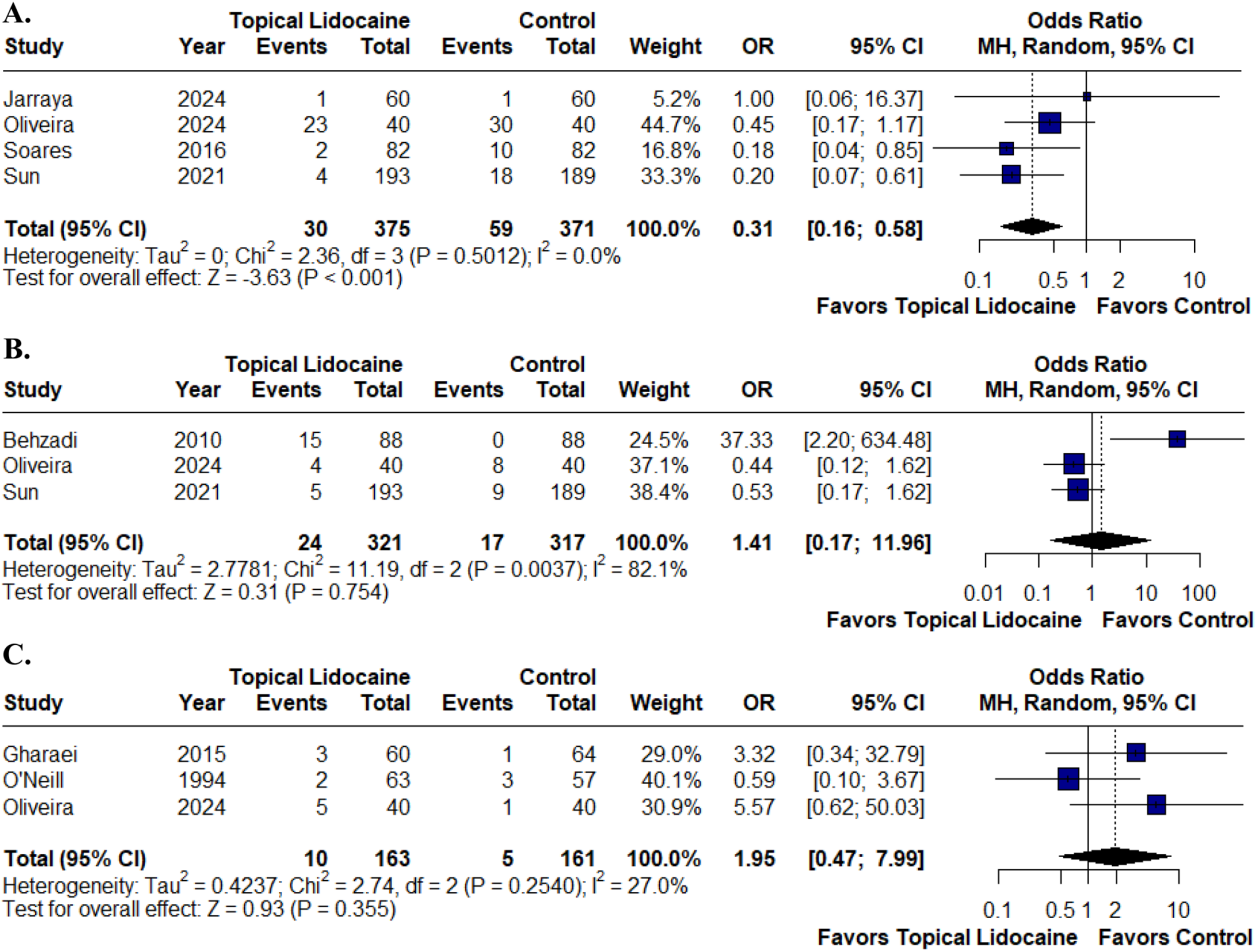
Topical lidocaine was associated with a significantly lower incidence of sore throat (A), but there was no significant reduction in the occurrence of hoarseness (B) or vomiting (C) compared with the control group.

In those receiving topical airway lidocaine, there was a statistically significant reduction in the incidence of sore throat compared with the control group (OR 0.31; 95% CI 0.16 to 0.58; *p* < 0.001; *I* ^2^ = 0%; Figure 4A). However, there was no statistically significant difference between the groups regarding hoarseness (OR 1.41; 95% CI 0.17 to 11.96; *p* = 0.754; *I* ^2^ = 82.1%; Figure 4B) and vomiting (OR 1.95; 95% CI 0.47 to 7.99; *p* = 0.355; *I* ^2^ = 27%; Figure 4C).

#### 3.2.4 Oxygenation and Vital Signs

**FIGURE 5:**
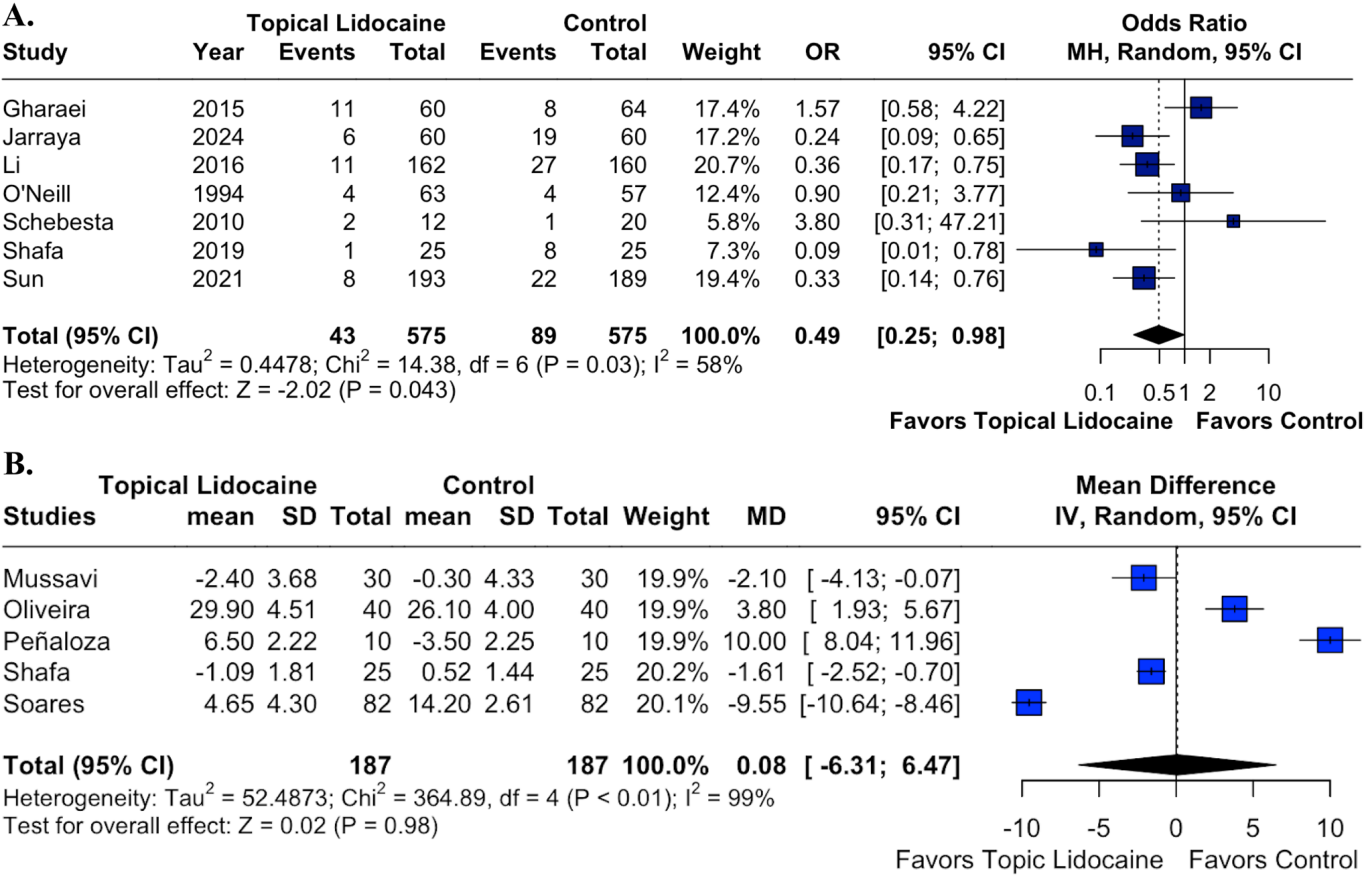
Topical lidocaine was associated with a significantly lower incidence of desaturation (A), but heart rate (B) was not significantly different between the groups.

The incidence of desaturation was evaluated in seven RCTs involving 1150 patients, demonstrating a statistically significant reduction in the topical lidocaine group compared with the control group (OR 0.49; 95% CI 0.25 to 0.98; *p* = 0.043; *I* ^2^ = 58%; Figure 5A). In contrast, there was no statistically significant difference between the groups regarding heart rate (beats/min) (MD 0.08; 95% CI −6.31 to 6.47; *p* = 0.98; *I* ^2^ = 99%; Figure 5B).

### 3.3 Subgroup and Supplementary Analyses

Subgroup analyses are summarized in Table S2. In supplementary analyses combining RCTs and observational data, there was no statistically significant difference between groups in terms of laryngospasm (OR 0.69; 95% CI 0.36 to 1.35; *p* = 0.284; *I* ^2^ = 77.8%; Figure S1A), cough (OR 0.76; 95% CI 0.41 to 1.41; *p* = 0.386; *I* ^2^ = 78.3%; Figure S1B), desaturation (OR 0.62; 95% CI 0.29 to 1.34; *p* = 0.223; *I* ^2^ = 80.2%; Figure S1C).

#### 3.3.1 Lidocaine Formulation

In a subgroup analysis based on lidocaine formulation, the gel/cream group showed no statistically significant difference between the topical lidocaine and control groups for laryngospasm (OR 0.33; 95% CI 0.04 to 2.70; *p* = 0.303; *I* ^2^ = 73.4%; Figure S2A), cough (OR 0.47; 95% CI 0.12 to 1.92; *p* = 0.294; *I* ^2^ = 86.3%; Figure S2B), and desaturation (OR 0.90; 95% CI 0.33 to 2.45; *p* = 0.834; *I* ^2^ = 60%; Figure S2C). For the nebulization subgroup, there was no statistically significant difference in the incidence of laryngospasm between groups (OR 0.43; 95% CI 0.12 to 1.61; *p* = 0.212; *I* ^2^ = 0%; Figure S2D). Lastly, in the local injection subgroup, there was no statistically significant difference between groups regarding cough (OR 0.97; 95% CI 0.58 to 1.63; *p* = 0.917; *I* ^2^ = 0.8%; Figure S2E).

#### 3.3.2 Airway Management

In a subgroup analysis focusing on airway management, patients who underwent endotracheal intubation or extubation showed no statistically significant difference between groups for laryngospasm (OR 0.49; 95% CI 0.21 to 1.14; *p* = 0.097; *I* ^2^ = 52.1%; Figure S3A) and cough (OR 0.67; 95% CI 0.31 to 1.44; *p* = 0.304; *I* ^2^ = 58.2%; Figure S3B). Likewise, in the laryngeal mask subgroup, there was no statistically significant difference between the groups in terms of laryngospasm (OR 0.33; 95% CI 0.04 to 2.70; *p* = 0.303; *I* ^2^ = 73.4%; Figure S3C), cough (OR 0.47; 95% CI 0.12 to 1.92; *p* = 0.294; *I* ^2^ = 86.3%; Figure S3D), and desaturation (OR 0.90; 95% CI 0.33 to 2.45; *p* = 0.834; *I* ^2^ = 60%; Figure S3E).

#### 3.3.3 Lidocaine Concentration

In a subgroup analysis of lidocaine concentration, both 1% lidocaine (OR 0.64; 95% CI 0.23 to 1.78; *p* = 0.391; *I* ^2^ = 0%; Figure S4A) and 2% lidocaine (OR 0.61; 95% CI 0.23 to 1.68; *p* = 0.342; *I* ^2^ = 82.4%; Figure S4B) showed no statistically significant difference between groups regarding cough. Likewise, the use of 2% lidocaine showed no statistically significant difference between groups regarding laryngospasm (OR 0.71; 95% CI 0.36 to 1.41; *p* = 0.331; *I* ^2^ = 44%; Figure S4C) and desaturation (OR 0.66; 95% CI 0.28 to 1.57; *p* = 0.351; *I* ^2^ = 64%; Figure S4D).

#### 3.3.4 Presence of Upper Respiratory Tract Infection

In a subgroup analysis based on the presence of URTI, no statistically significant difference was found between the groups regarding desaturation (OR 0.66; 95% CI 0.20 to 2.18; *p* = 0.495; *I* ^2^ = 71%; Figure S5).

#### 3.3.5 Children's Age

In a subgroup analysis by age, children in the preschool group exhibited no statistically significant difference between groups for laryngospasm (OR 0.34; 95% CI 0.10 to 1.10; *p* = 0.073; *I* ^2^ = 63.3%; Figure S6A), cough (OR 0.43; 95% CI 0.15 to 1.28; *p* = 0.130; *I* ^2^ = 83.1%; Figure S6B), and desaturation (OR 0.56; 95% CI 0.28 to 1.11; *p* = 0.097; *I* ^2^ = 58.6%; Figure S6C). Similarly, there was no statistically significant difference in the incidence of laryngospasm (OR 0.67; 95% CI 0.24 to 1.86; *p* = 0.443; *I* ^2^ = 56.7%; Figure S6D) and cough (OR 0.95; 95% CI 0.53 to 1.71; *p* = 0.869; *I* ^2^ = 0.8%; Figure S6E) for the school‐aged group.

### 3.4 Risk of Bias and Certainty of Evidence

According to the RoB‐2 tool, eight randomized studies were classified as having a low overall risk of bias [7, 8, 9, 30, 31, 32, 36, 38], while three were labeled with some concerns [6, 33, 34], particularly related to bias from deviations from the intended intervention. In contrast, the trials conducted by O'Neill et al., Peñaloza et al., and Staffel et al. were classified as having a high overall risk of bias due to deviations in the randomization process and blinding (D1) [14, 35, 37]. When assessed with the ROBINS‐I tool, two observational studies were deemed to pose moderate risks [39, 41], while another was identified as having critical bias risk [40]. Specifically, the cohort by Hamilton et al. was rated as having a serious risk due to unmeasured or poorly measured confounding variables [40]. These evaluations are illustrated in the Figures S7 and S8. According to the GRADE criteria, the quality of evidence was rated as high for the outcome of desaturation, and as moderate for the outcomes of laryngospasm and cough (Table S3).

### 3.5 Sensitivity Analyses

Leave‐one‐out sensitivity analyses were performed for the laryngospasm, cough, and desaturation outcomes. Studies were sequentially removed, and the data were re‐analyzed to ensure the stability of the overall effects. Research dominance was assigned whenever pooled effect size *p*‐values changed from significant to non‐significant or vice versa when removing the study [13]. Significant changes were observed in the laryngospasm outcome when the trials by Li et al., Sun et al., or Peñaloza et al. were omitted (Figure S9A) [6, 8, 37]. Moreover, the incidence of cough changed from non‐significant to significant when the Gharaei et al. trial was individually removed (Figure S9B) [33]. Finally, the significance of desaturation shifted to non‐significant when the studies by Gharaei et al., Schebesta et al., or O'Neil et al. were omitted (Figure S9C) [7, 14, 33]. These results are available in Figure S9 of the Supporting Information Appendix.

#### 3.5.1 Correlation Analyses

Meta‐regression analyses are presented in the Figures S10 and S11. In the context of a mixed‐effects meta‐analysis for the outcome of cough, significant residual heterogeneity was observed, with *I* ^2^ consistently exceeding 60%. Nevertheless, meta‐regression analyses revealed that none of the assessed moderators significantly influenced intervention efficacy. Specifically, age had a non‐significant effect (estimate = 0.0158, *p* = 0.9612), as did sample size (estimate = −0.0045, *p* = 0.1748) and type of device (LMA or IOT, estimate = 0.3192, *p* = 0.6762). Similarly, the type of lidocaine administration—whether nebulization, gel, or local injection—was also not associated with significant differences in effect sizes (*p* > 0.05 for all). Amongst these methods, local injection showed a positive but non‐significant tendency (estimate = 1.1316, *p* = 0.3477), while nebulization demonstrated a slight negative trend with no significant effect (estimate = −0.4836, *p* = 0.8174).

Notably, when comparing cough outcomes across different control groups, both saline solution and no intervention resulted in a significantly lower effect size compared to intravenous lidocaine (estimate = −1.5772, *p* < 0.0001). The variance explained by the moderators was minimal across all models, indicating they did not sufficiently account for the observed heterogeneity in effect sizes. For laryngospasm outcomes, the heterogeneity was lower compared to cough, with *I* ^2^ below 50% in most models, indicating less variability amongst studies. The results indicated no significant associations for age (estimate = 0.2074, *p* = 0.3500) or sample size (estimate = −0.0045, *p* = 0.1748). Similarly, the type of device (LMA or IOT, estimate = 0.0846, *p* = 0.9230) and lidocaine administration type (nebulization, gel, or local injection) were not associated with significant differences in effect sizes (*p* > 0.05 for all). Consistent with the cough analysis, the variance explained by moderators was minimal across all models, indicating that the included variables did not sufficiently account for the observed heterogeneity in effect sizes.

## 4 Discussion

In this systematic review and meta‐analysis involving 14 RCTs and a total of 1937 patients, we evaluated the effectiveness of topical lidocaine in preventing PRAEs in children undergoing airway management. The key findings associated with lidocaine topicalization are as follows: (1) a significant decrease in laryngospasm, desaturation, and sore throat occurrences linked to topical lidocaine anesthesia for airway manipulation in the pooled analysis; (2) no significant benefit of topical airway lidocaine in the incidence of bronchospasm, cough, severe cough, hoarseness, vomiting, and heart rate changes in the overall analysis.

Airway management is essential for patients receiving anesthesia during surgical or diagnostic procedures, and it is particularly crucial in life‐threatening scenarios such as cardiopulmonary resuscitation and critical care [42]. The recently published guidelines from the joint European Society of Anaesthesiology and Intensive Care and the British Journal of Anesthesia (ESAIC‐BJA) offer comprehensive recommendations to assist clinicians in integrating the most current evidence regarding the neonatal and infant management of normal, expected, and unexpected difficult airways, from initial assessment to extubation [4, 43]. Nonetheless, these guidelines are inherently limited due to a lack of supporting evidence across various domains of airway management, often relying on expert opinions or case studies instead of robust clinical trials [44].

The use of topical laryngeal lidocaine is a common practice in pediatric anesthesia. It is typically employed to facilitate open airway procedures, assist with tracheal intubation, or minimize the occurrence of PRAEs such as coughing and laryngospasm [45]. However, there is insufficient evidence regarding the impact of topical lidocaine on the sensitivity of upper airway reflexes and swallowing, the duration for which airway reflexes are diminished, and the optimal and safe maximum dosage when administered via this route [46].

Some randomized studies have reported that lidocaine topicalization of the airways is correlated with improved clinical outcomes in the perioperative and recovery timeframes. However, this association has not been consistently demonstrated across all available research [6, 8, 14, 30, 35, 38]. In the trials by Sun et al. and Li et al., both nebulized and cream formulations of lidocaine were effective in attenuating cough and laryngospasm reflexes by their local anesthetic effects on the upper airway mucosa, as well as their anti‐inflammatory and bronchodilator properties, which helped children tolerate airway manipulation [6, 8]. In contrast, other RCTs, such as those conducted by Oliveira et al. and Soares et al., found no significant benefits of tracheal tube cuffs filled with lidocaine in preventing laryngotracheal complications in pediatric patients undergoing general anesthesia [3, 7, 9, 31, 33, 35, 37].

In clinical anaesthesiology practice, airway topicalization is typically based on patient characteristics, procedural requirements, and institutional protocols [42]. Despite the positive findings in our pooled analysis, subgroup analyses did not reveal consistent efficacy of topical lidocaine across different formulations, concentrations, routes of administration, airway devices, presence of URTI, or patient age groups. These results underscore that the benefits of topical lidocaine may be context‐dependent and highlight the heterogeneity of the available evidence. Therefore, further high‐quality research is needed to identify which patients and clinical contexts derive the greatest benefit from this intervention.

It is important to acknowledge that the clinical rationale for using topical lidocaine may involve accepting minor, transient complications during application in order to reduce the risk of more consequential airway events during subsequent manipulation. This distinction underscores that clinicians may reasonably prioritize preventing adverse events at critical procedural stages over avoiding transient effects during drug administration. Notably, these considerations align with the recent consensus recommendations for pediatric airway topicalization using lidocaine, which emphasize careful risk–benefit assessment and reinforce the ongoing need for high‐quality research to address persistent knowledge gaps in pediatric airway management [5].

This study represents a comprehensive analysis of the safety and efficacy of lidocaine topicalization in pediatric patients undergoing airway management. Although a prior systematic review and meta‐analysis reported a statistical difference between topical and intravenous administration strategies, it was limited by a smaller patient sample and focused primarily on studies that examined the incidence of perioperative and recovery‐related laryngospasm. The review also suffered from a limited control group for comparison, which was restricted to intravenous versus topical airway lidocaine [10, 11].

### 4.1 Limitations

Our study has important limitations. First, there is considerable heterogeneity in the lidocaine regimens used, including differences in doses (with 1% being the most common), administration techniques, airway management devices—most frequently endotracheal intubation—and children's ages. These differences contribute to the overall heterogeneity observed in our analysis. Although we performed subgroup analyses for these covariates, we cannot exclude the possibility that these evaluations may be underpowered due to the small number of studies in each sub‐analysis. Additionally, substantial variability existed regarding the timing of PRAEs reporting across studies, as well as a lack of standardization in the definitions of desaturation and laryngospasm, which may introduce bias into the analyses of these outcomes.

Finally, this meta‐analysis could not adequately address pediatric patients with high‐risk factors for PRAEs, such as asthma, recent URTI, allergic rhinitis, eczema, nocturnal dry cough, and passive smoking [47]. These conditions are well known to increase susceptibility to airway complications during anesthesia due to laryngeal hyperreactivity [48, 49], and therefore our findings cannot be extrapolated to these populations. This highlights the need for further research focused on high‐risk pediatric patients to better inform clinical decision‐making and optimize perioperative airway management.

## 5 Conclusion

The findings from this systematic review and meta‐analysis suggest that topical lidocaine may reduce the incidence of undesirable airway reflexes such as laryngospasm, desaturation, and sore throat in children undergoing airway management. However, its benefit for other PRAEs requires further investigation, especially in high‐risk populations, to elucidate the role of lidocaine topicalization in pediatric airway management.

## Author Contributions

Conception and design of the research: Gabriel Soares de Sousa and Elizabet Taylor Pimenta Weba. Acquisition of data: Alexandros Páris de Mesquita Ipácio, Elizabet Taylor Pimenta Weba, and Rafaela Machado Filardi. Analysis and interpretation of the data: Rafaela Machado Filardi, Alexandros Páris de Mesquita Ipácio, Christian Ken Fukunaga, Rafael Andrade Sampaio Silva, Marco Antonio Figueiredo Teixeira, and Elizabet Taylor Pimenta Weba. Writing of the first manuscript draft: Elizabet Taylor Pimenta Weba, Rafael Andrade Sampaio Silva, Alexandros Páris de Mesquita Ipácio, and Christian Ken Fukunaga. Critical revision of the manuscript for intellectual content: All authors.

## Ethics Statement

The authors have nothing to report.

## Consent

The authors have nothing to report.

## Conflicts of Interest

Britta S. von Ungern‐Sternberg is a section editor for Pediatric Anesthesia. None of the other authors has any conflicts of interest to disclose.

## Supporting information


**Figure S1:** Supplementary analysis of RCTs and observational studies assessing the association between topical lidocaine and the incidence of laryngospasm (A), cough (B), and desaturation (C).
**Figure S2:** Subgroup analysis of the associations between lidocaine formulations and PRAEs. This includes gel/cream lidocaine with laryngospasm (A), cough (B), and desaturation (C); nebulized lidocaine with laryngospasm (D); and local lidocaine injection with cough (E).
**Figure S3:** Subgroup analysis of the associations between airway management devices and PRAEs. This includes intubation/extubation procedures with laryngospasm (A) and cough (B); as well as laryngeal mask airway procedures with laryngospasm (C), cough (D) and desaturation (E).
**Figure S4:** Subgroup analysis of the associations between lidocaine concentrations and PRAEs. This includes 1% lidocaine with cough (A); and 2% lidocaine with cough (B), laryngospasm (C), and desaturation (D).
**Figure S5:** Subgroup analysis assessing the association between preoperative upper respiratory infection and the incidence of desaturation.
**Figure S6:** Subgroup analysis of the associations between paediatric patients' age and PRAEs. This includes preschool‐aged children with laryngospasm (A), cough (B), and desaturation (C); as well as school‐aged children with laryngospasm (D) and cough (E).
**Figure S7:** Risk of bias in randomized trials with the RoB‐2 tool, illustrated in a summary plot (A) and a traffic light plot (B).
**Figure S8:** Risk of bias in non‐randomized cohorts with the ROBINS‐I tool, illustrated in a summary plot (A) and a traffic light plot (B).
**Figure S9:** Leave‐one‐out sensitivity analyses for the primary outcomes of laryngospasm (A), cough (B) and desaturation (C).
**Figure S10:** Meta‐regression sensitivity analyses of laryngospasm with potential moderators, including age (A), sample size (B), control groups (C), airway management devices (D), and type of lidocaine administration (E).
**Figure S11:** Meta‐regression sensitivity analyses of cough with potential moderators, including age (A), sample size (B), control groups (C), airway management devices (D), and type of lidocaine administration (E).
**Table S1:** Baseline characteristics of observational studies included.
**Table S2:** Summary of subgroup analyses findings.
**Table S3:** GRADE approach to ascertain certainty of evidence.

## Data Availability

This meta‐analysis used data from previously published studies; therefore, all data and study materials are in the public domain. The meta‐analysis authors do not have patient‐level data from the individual studies.
