# Supplementary material for: Topical Lidocaine During Airway Manipulation in Pediatric Anesthesia: A Systematic Review and Meta‐Analysis
Source: Paediatr Anaesth. 2025 Nov 11;36(2):151–63. doi: 10.1111/pan.70077 (PMC12779226; doi:10.1111/pan.70077)
Supplement: Supplementary file 1 — Figure S1: Supplementary analysis of RCTs and observational studies assessing the association between topical lidocaine and the incidence of laryngospasm (A), cough (B), and desaturation (C). Figure S2: Subgroup analysis of the associations between lidocaine formulations and PRAEs. This includes gel/cream lidocaine with laryngospasm (A), cough (B), and desaturation (C); nebulized lidocaine with laryngospasm (D); and local lidocaine injection with cough (E). Figure S3: Subgroup analysis of the associations between airway management devices and PRAEs. This includes intubation/extubation procedures with laryngospasm (A) and cough (B); as well as laryngeal mask airway procedures with laryngospasm (C), cough (D) and desaturation (E). Figure S4: Subgroup analysis of the associations between lidocaine concentrations and PRAEs. This includes 1% lidocaine with cough (A); and 2% lidocaine with cough (B), laryngospasm (C), and desaturation (D). Figure S5: Subgroup analysis assessing the association between preoperative upper respiratory infection and the incidence of desaturation. Figure S6: Subgroup analysis of the associations between paediatric patients' age and PRAEs. This includes preschool‐aged children with laryngospasm (A), cough (B), and desaturation (C); as well as school‐aged children with laryngospasm (D) and cough (E). Figure S7: Risk of bias in randomized trials with the RoB‐2 tool, illustrated in a summary plot (A) and a traffic light plot (B). Figure S8: Risk of bias in non‐randomized cohorts with the ROBINS‐I tool, illustrated in a summary plot (A) and a traffic light plot (B). Figure S9: Leave‐one‐out sensitivity analyses for the primary outcomes of laryngospasm (A), cough (B) and desaturation (C). Figure S10: Meta‐regression sensitivity analyses of laryngospasm with potential moderators, including age (A), sample size (B), control groups (C), airway management devices (D), and type of lidocaine administration (E). Figure S11: Meta‐regression sensitivity anal [file PAN-36-151-s001.pdf]

Complete and adapted search strategy for PubMed and Embase databases: ('children'/exp OR children OR 'child'/exp OR child OR 'pediatric'/exp OR pediatric OR 'paediatric'/exp OR paediatric OR 'adolescent'/exp OR adolescent OR 'infant'/exp OR infant OR 'newborn'/exp OR newborn OR 'neonate'/exp OR neonate) AND ('topical airway anesthesia' OR 'airway topicalization' OR 'airway topicalisation' OR 'local airway anesthesia' OR 'mucosal anesthesia' OR 'topical anesthesia'/exp OR 'topical anesthesia' OR 'topical local anesthetic' OR 'topical pharyngeal' OR 'topical lidocaine' OR tal OR 'local lidocaine' OR 'lidocaine spray' OR 'nebulized lidocaine' OR 'nebulised lidocaine') AND ('laryngospasm'/exp OR laryngospasm OR 'laryngismus'/exp OR laryngismus OR 'cough'/exp OR cough OR 'bronchospasm'/exp OR bronchospasm OR 'bronchial spasm'/exp OR 'bronchial spasm' OR 'desaturation'/exp OR desaturation OR bucking OR 'respiratory'/exp OR respiratory OR 'airway'/exp OR airway OR 'obstruction'/exp OR obstruction OR 'stridor'/exp OR stridor OR 'intubation'/exp OR intubation OR 'extubation'/exp OR extubation OR 'reintubation'/exp OR reintubation OR 'oxygen'/exp OR oxygen OR 'aspiration'/exp OR aspiration OR 'breath holding'/exp OR 'breath holding' OR 'apnea'/exp OR apnea OR 'hypopnea'/exp OR hypopnea)

Complete and adapted search strategy for Cochrane database: (Children OR Child OR pediatric OR paediatric OR adolescent OR infant OR newborn OR neonate) AND ("topical airway anesthesia" OR “airway topicalization” OR “airway topicalisation” OR “local airway anesthesia” OR “mucosal anesthesia” OR “topical anesthesia” OR “topical local anesthetic” OR “topical pharyngeal” OR “topical lidocaine” OR TAL OR “local lidocaine” OR “lidocaine spray” OR “nebulized lidocaine” OR “nebulised lidocaine”) AND (Laryngospasm OR Laryngismus OR cough OR bronchospasm OR “bronchial spasm” OR desaturation OR bucking OR respiratory OR airway OR obstruction OR stridor OR intubation OR extubation OR reintubation OR oxygen OR aspiration OR “breath holding” OR apnea OR hypopnea)

Supplementary Figure 1. Supplementary analysis of RCTs and observational studies assessing the association between topical lidocaine and the incidence of laryngospasm (A), cough (B), and desaturation (C).

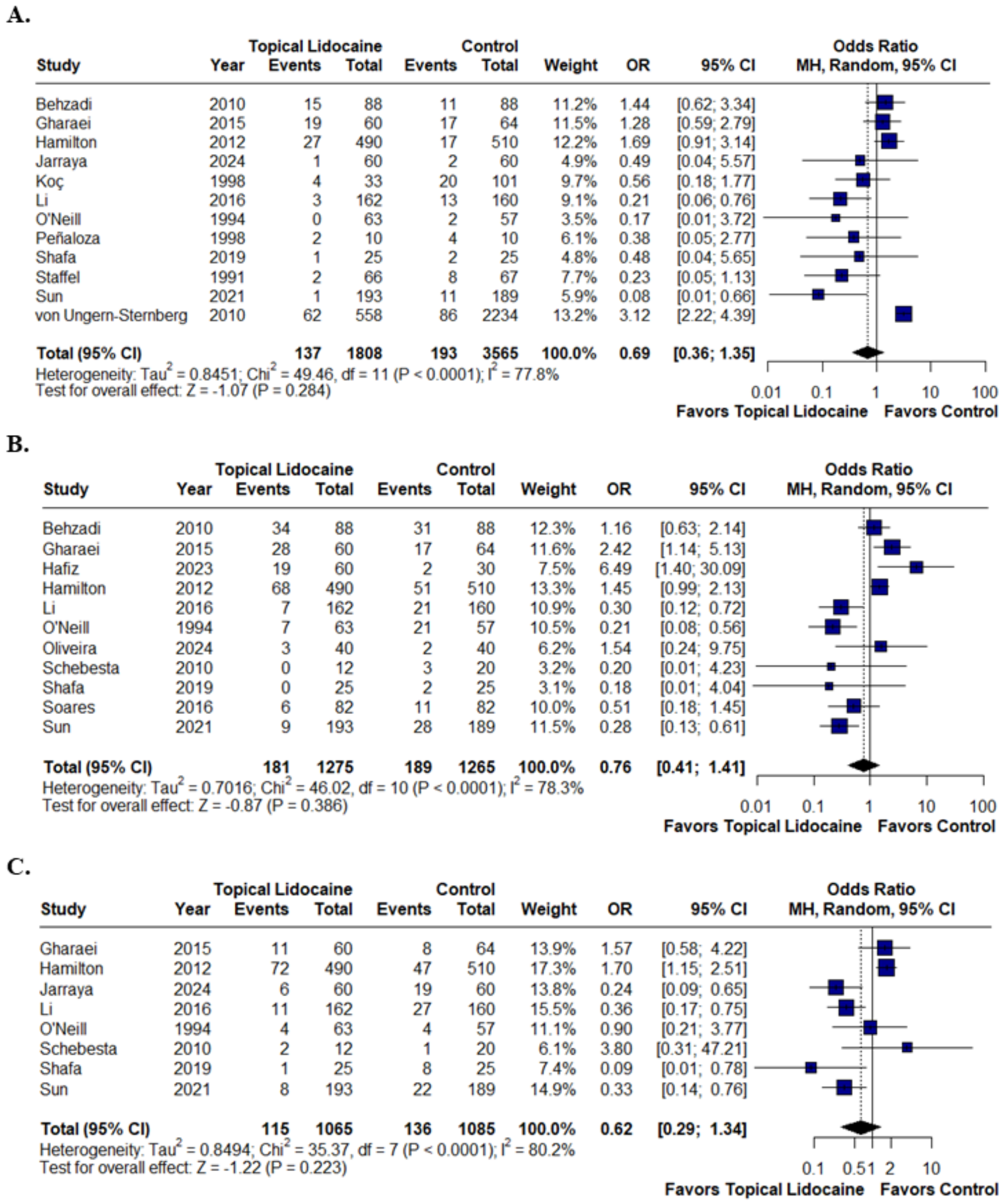

Supplementary Figure 2. Subgroup analysis of the associations between lidocaine formulations and PRAEs. This includes gel/cream lidocaine with laryngospasm (A), cough (B), and desaturation (C); nebulised lidocaine with laryngospasm (D); and local lidocaine injection with cough (E).

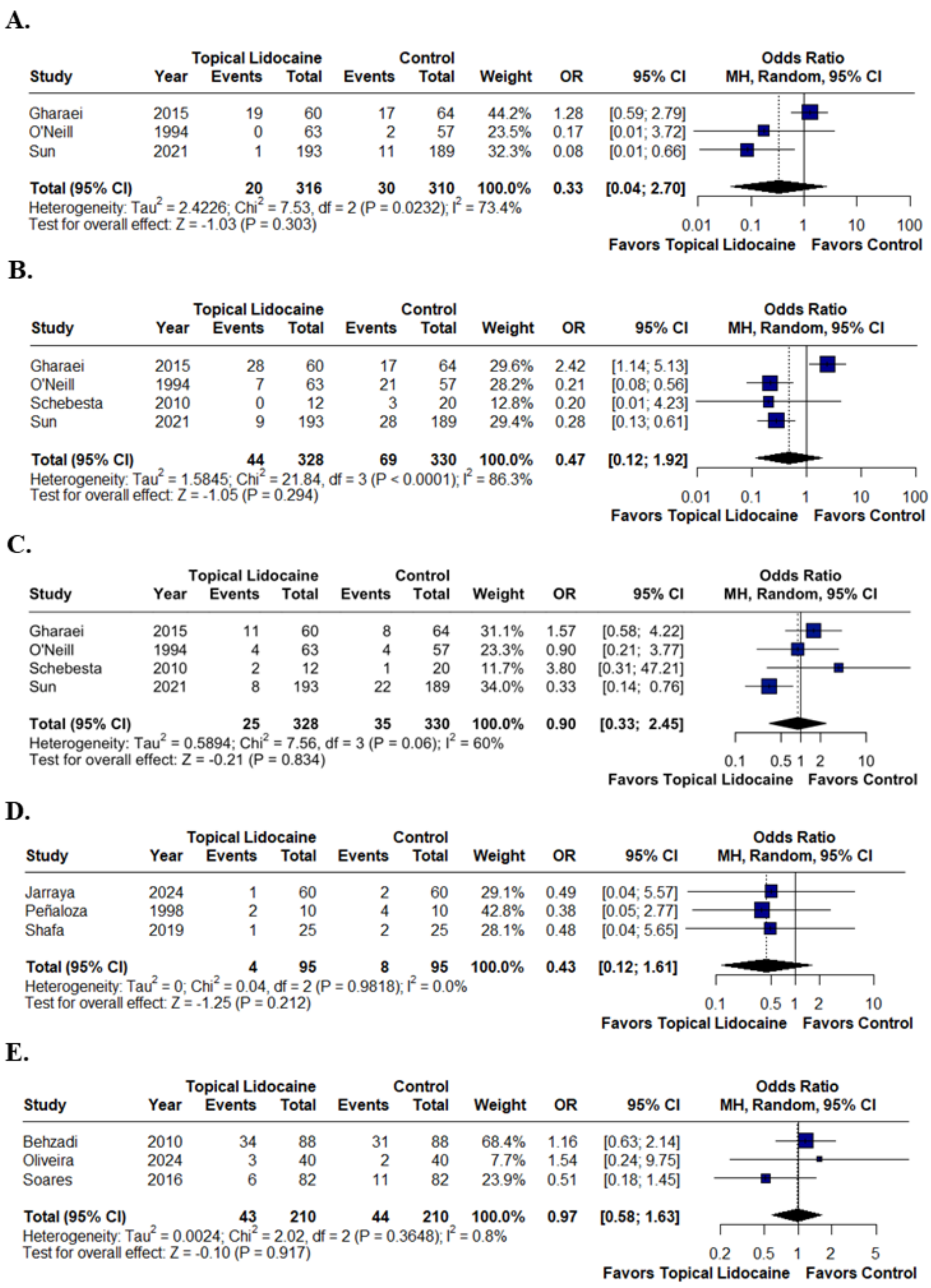

Supplementary Figure 3. Subgroup analysis of the associations between airway management devices and PRAEs. This includes intubation/extubation procedures with laryngospasm (A) and cough (B); as well as laryngeal mask airway procedures with laryngospasm (C), cough (D) and desaturation (E).

A.

Study	Year	Topical Lidocaine Events	Topical Lidocaine Total	Control Events	Control Total	Weight	OR	95% CI
Behzadi	2010	15	88	11	88	28.4%	1.44	[0.62; 3.34]
Koç	1998	4	33	20	101	22.6%	0.56	[0.18; 1.77]
Li	2016	3	162	13	160	20.6%	0.21	[0.06; 0.76]
Peñaloza	1998	2	10	4	10	12.1%	0.38	[0.05; 2.77]
Staffel	1991	2	66	8	67	16.3%	0.23	[0.05; 1.13]
**Total (95% CI)**		**26**	**359**	**56**	**426**	**100.0%**	**0.49**	**[0.21; 1.14]**

Heterogeneity: $Tau^2 = 0.4522$; $Chi^2 = 8.35$, df = 4 (P = 0.0797); $I^2 = 52.1\%$
Test for overall effect: Z = -1.66 (P = 0.097)

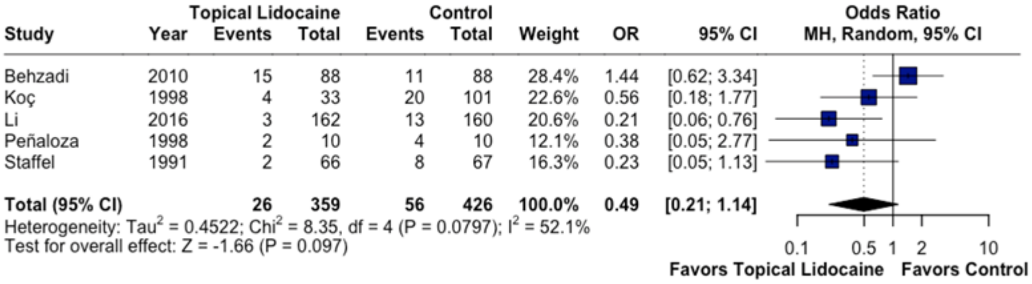

B.

Study	Year	Topical Lidocaine Events	Topical Lidocaine Total	Control Events	Control Total	Weight	OR	95% CI
Behzadi	2010	34	88	31	88	35.1%	1.16	[0.63; 2.14]
Li	2016	7	162	21	160	28.1%	0.30	[0.12; 0.72]
Oliveira	2024	3	40	2	40	12.4%	1.54	[0.24; 9.75]
Soares	2016	6	82	11	82	24.4%	0.51	[0.18; 1.45]
**Total (95% CI)**		**50**	**372**	**65**	**370**	**100.0%**	**0.67**	**[0.31; 1.44]**

Heterogeneity: $Tau^2 = 0.3328$; $Chi^2 = 7.18$, df = 3 (P = 0.0662); $I^2 = 58.2\%$
Test for overall effect: Z = -1.03 (P = 0.304)

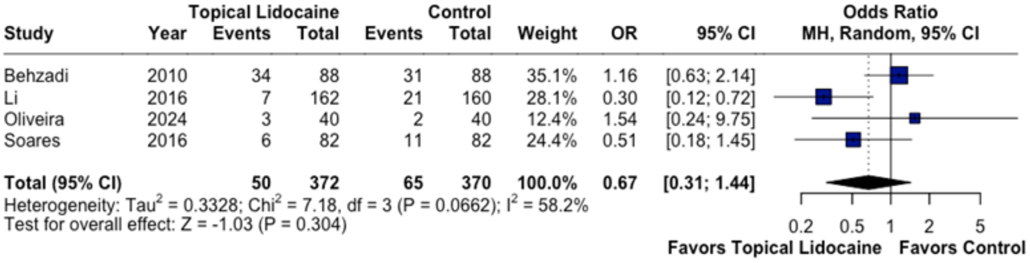

C.

Study	Year	Topical Lidocaine Events	Topical Lidocaine Total	Control Events	Control Total	Weight	OR	95% CI
Gharaei	2015	19	60	17	64	44.2%	1.28	[0.59; 2.79]
O'Neill Non-Morphine	1994	0	63	2	57	23.5%	0.17	[0.01; 3.72]
Sun	2021	1	193	11	189	32.3%	0.08	[0.01; 0.66]
**Total (95% CI)**		**20**	**316**	**30**	**310**	**100.0%**	**0.33**	**[0.04; 2.70]**

Heterogeneity: $Tau^2 = 2.4226$; $Chi^2 = 7.53$, df = 2 (P = 0.0232); $I^2 = 73.4\%$
Test for overall effect: Z = -1.03 (P = 0.303)

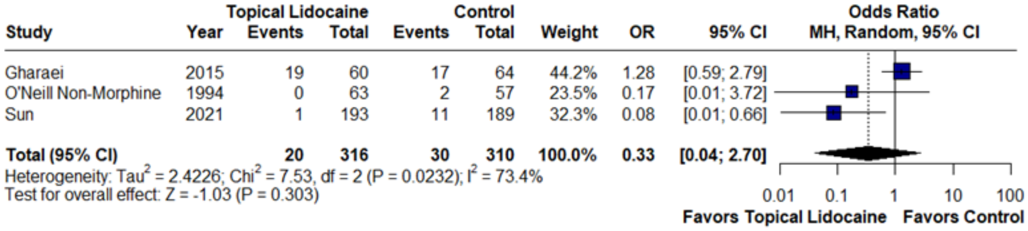

D.

Study	Year	Topical Lidocaine Events	Topical Lidocaine Total	Control Events	Control Total	Weight	OR	95% CI
Gharaei	2015	28	60	17	64	29.6%	2.42	[1.14; 5.13]
O'Neill	1994	7	63	21	57	28.2%	0.21	[0.08; 0.56]
Schebesta	2010	0	12	3	20	12.8%	0.20	[0.01; 4.23]
Sun	2021	9	193	28	189	29.4%	0.28	[0.13; 0.61]
**Total (95% CI)**		**44**	**328**	**69**	**330**	**100.0%**	**0.47**	**[0.12; 1.92]**

Heterogeneity: $Tau^2 = 1.5845$; $Chi^2 = 21.84$, df = 3 (P < 0.0001); $I^2 = 86.3\%$
Test for overall effect: Z = -1.05 (P = 0.294)

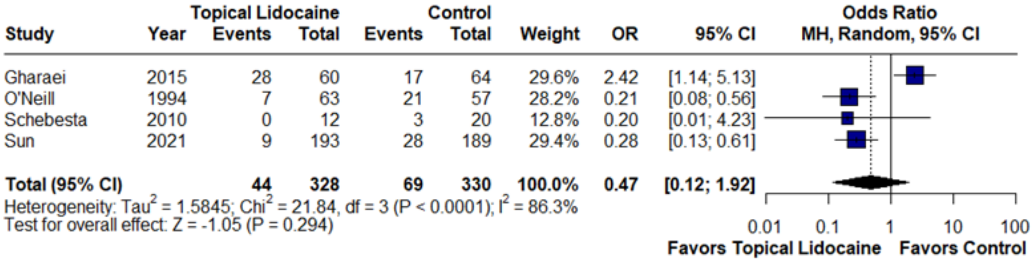

E.

Study	Year	Topical Lidocaine Events	Topical Lidocaine Total	Control Events	Control Total	Weight	OR	95% CI
Gharaei	2015	11	60	8	64	31.1%	1.57	[0.58; 4.22]
O'Neill	1994	4	63	4	57	23.3%	0.90	[0.21; 3.77]
Schebesta	2010	2	12	1	20	11.7%	3.80	[0.31; 47.21]
Sun	2021	8	193	22	189	34.0%	0.33	[0.14; 0.76]
**Total (95% CI)**		**25**	**328**	**35**	**330**	**100.0%**	**0.90**	**[0.33; 2.45]**

Heterogeneity: $Tau^2 = 0.5894$; $Chi^2 = 7.56$, df = 3 (P = 0.06); $I^2 = 60\%$
Test for overall effect: Z = -0.21 (P = 0.834)

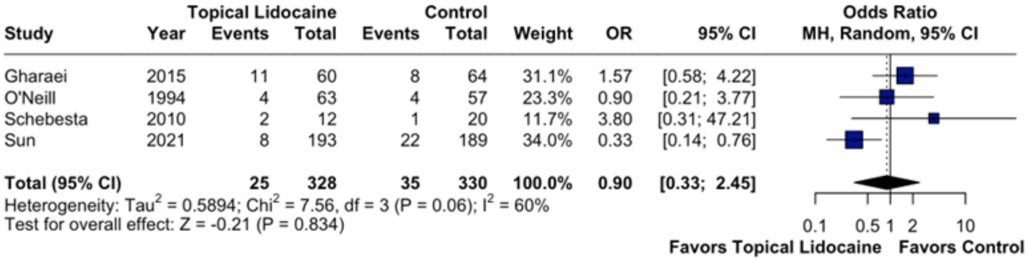

Supplementary Figure 4. Subgroup analysis of the associations between lidocaine concentrations and PRAEs. This includes 1% lidocaine with cough (A); and 2% lidocaine with cough (B), laryngospasm (C), and desaturation (D).

A.

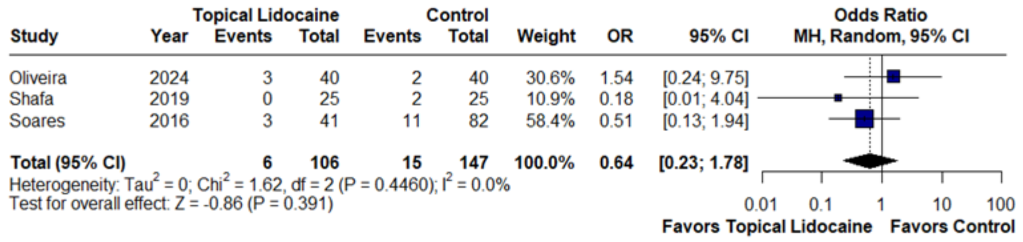

B.

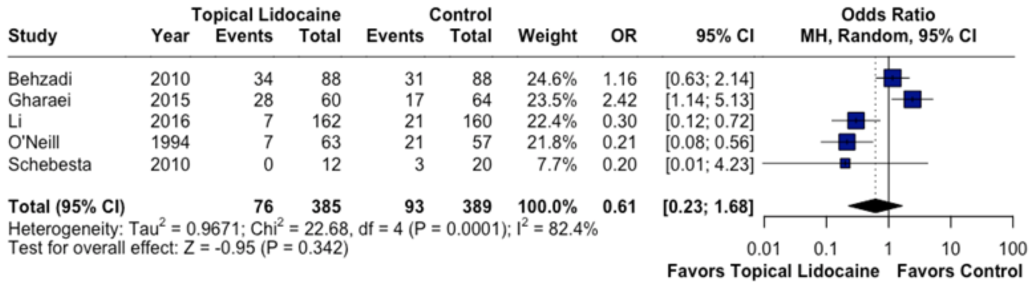

C.

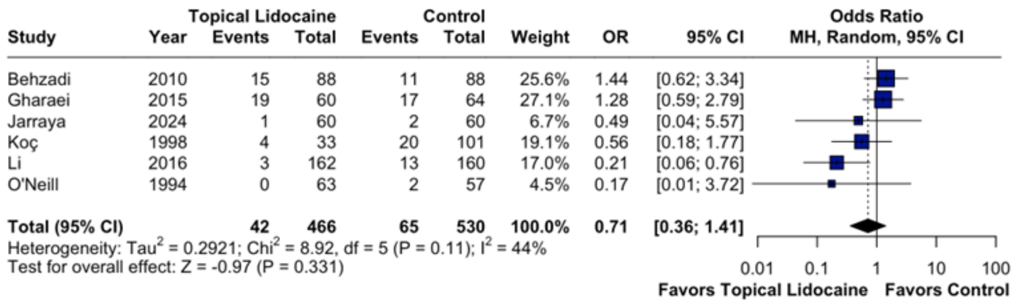

D.

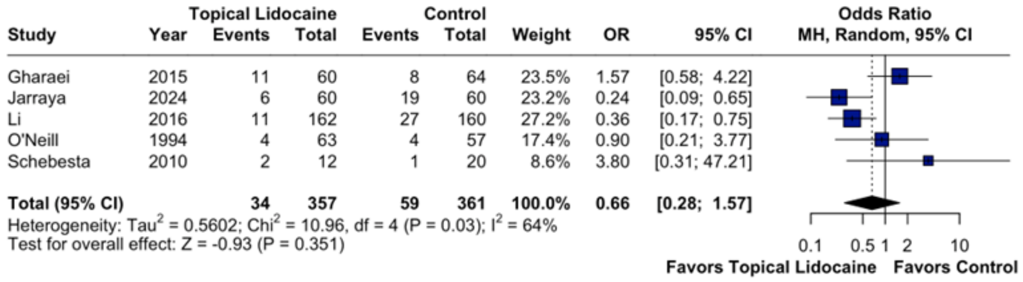

Supplementary Figure 5. Subgroup analysis assessing the association between preoperative upper respiratory infection and the incidence of desaturation.

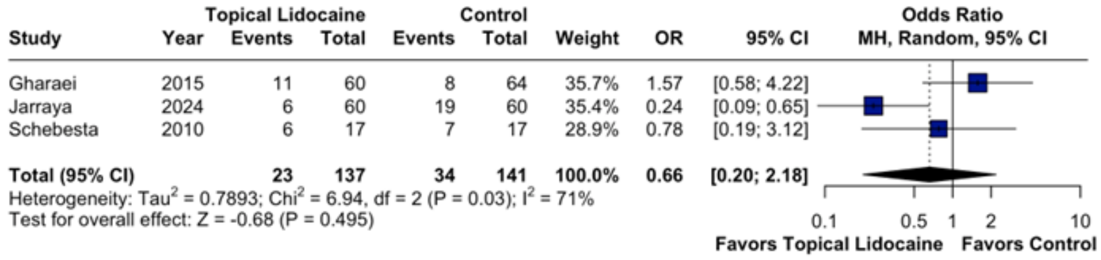

Supplementary Figure 6. Subgroup analysis of the associations between paediatric patients' age and PRAEs. This includes preschool-aged children with laryngospasm (A), cough (B), and desaturation (C); as well as school-aged children with laryngospasm (D) and cough (E).

A.

Study	Year	Topical Lidocaine Events	Topical Lidocaine Total	Control Events	Control Total	Weight	OR	95% CI
Gharaei	2015	19	60	17	64	30.3%	1.28	[0.59; 2.79]
Li	2016	3	162	13	160	24.8%	0.21	[0.06; 0.76]
O'Neill	1994	0	63	2	57	10.4%	0.17	[0.01; 3.72]
Peñaloza	1998	2	10	4	10	17.4%	0.38	[0.05; 2.77]
Sun	2021	1	193	11	189	16.9%	0.08	[0.01; 0.66]
**Total (95% CI)**		**25**	**488**	**47**	**480**	**100.0%**	**0.34**	**[0.10; 1.10]**

Heterogeneity: $Tau^2 = 1.0380$; $Chi^2 = 10.90$, df = 4 (P = 0.0278); $I^2 = 63.3\%$
Test for overall effect: Z = -1.79 (P = 0.073)

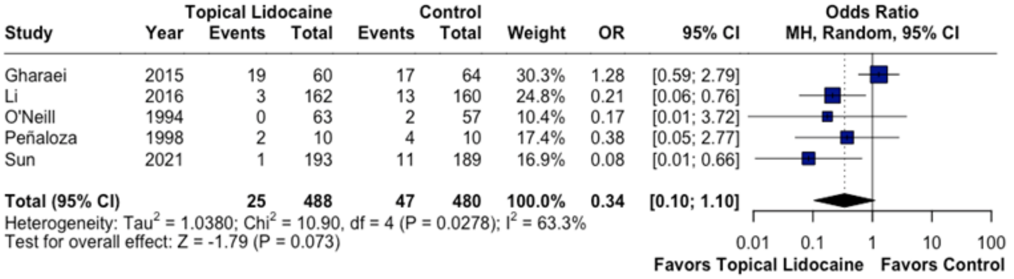

B.

Study	Year	Topical Lidocaine Events	Topical Lidocaine Total	Control Events	Control Total	Weight	OR	95% CI
Gharaei	2015	28	60	17	64	23.5%	2.42	[1.14; 5.13]
Li	2016	7	162	21	160	22.5%	0.30	[0.12; 0.72]
O'Neill	1994	7	63	21	57	22.0%	0.21	[0.08; 0.56]
Schebesta	2010	0	12	3	20	8.6%	0.20	[0.01; 4.23]
Sun	2021	9	193	28	189	23.3%	0.28	[0.13; 0.61]
**Total (95% CI)**		**51**	**490**	**90**	**490**	**100.0%**	**0.43**	**[0.15; 1.28]**

Heterogeneity: $Tau^2 = 1.1565$; $Chi^2 = 23.66$, df = 4 (P < 0.0001); $I^2 = 83.1\%$
Test for overall effect: Z = -1.51 (P = 0.130)

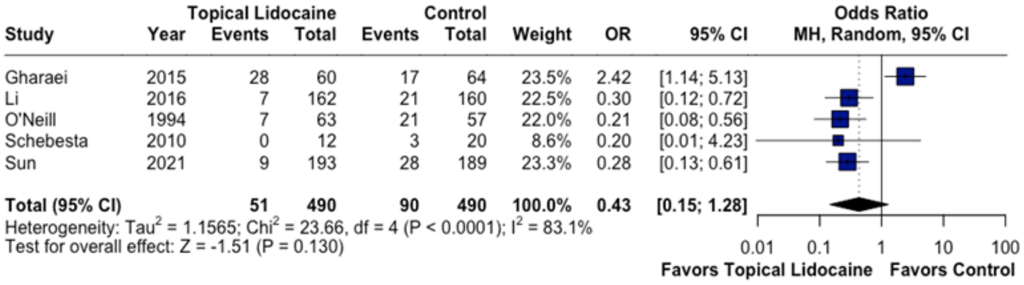

C.

Study	Year	Topical Lidocaine Events	Topical Lidocaine Total	Control Events	Control Total	Weight	OR	95% CI
Gharaei	2015	11	60	8	64	18.7%	1.57	[0.58; 4.22]
Jarraya	2024	6	60	19	60	18.5%	0.24	[0.09; 0.65]
Li	2016	11	162	27	160	22.6%	0.36	[0.17; 0.75]
O'Neill	1994	4	63	4	57	13.1%	0.90	[0.21; 3.77]
Schebesta	2010	2	12	1	20	6.0%	3.80	[0.31; 47.21]
Sun	2021	8	193	22	189	21.1%	0.33	[0.14; 0.76]
**Total (95% CI)**		**42**	**550**	**81**	**550**	**100.0%**	**0.56**	**[0.28; 1.11]**

Heterogeneity: $Tau^2 = 0.3987$; $Chi^2 = 12.08$, df = 5 (P = 0.0337); $I^2 = 58.6\%$
Test for overall effect: Z = -1.66 (P = 0.097)

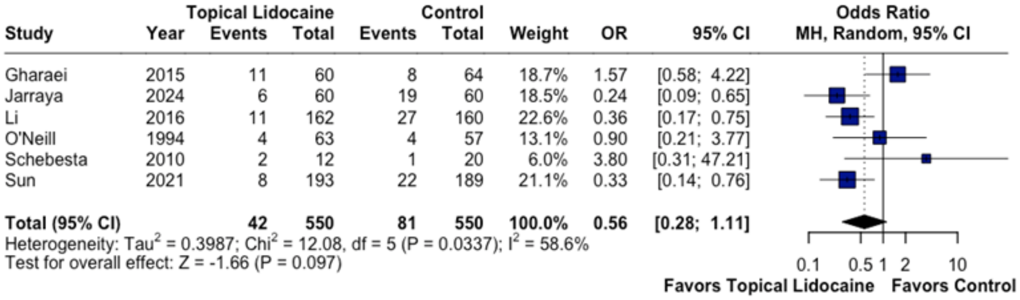

D.

Study	Year	Topical Lidocaine Events	Topical Lidocaine Total	Control Events	Control Total	Weight	OR	95% CI
Behzadi	2010	15	88	11	88	42.2%	1.44	[0.62; 3.34]
Koç	1998	4	33	20	101	33.6%	0.56	[0.18; 1.77]
Staffel	1991	2	66	8	67	24.2%	0.23	[0.05; 1.13]
**Total (95% CI)**		**21**	**187**	**39**	**256**	**100.0%**	**0.67**	**[0.24; 1.86]**

Heterogeneity: $Tau^2 = 0.4556$; $Chi^2 = 4.62$, df = 2 (P = 0.0992); $I^2 = 56.7\%$
Test for overall effect: Z = -0.77 (P = 0.443)

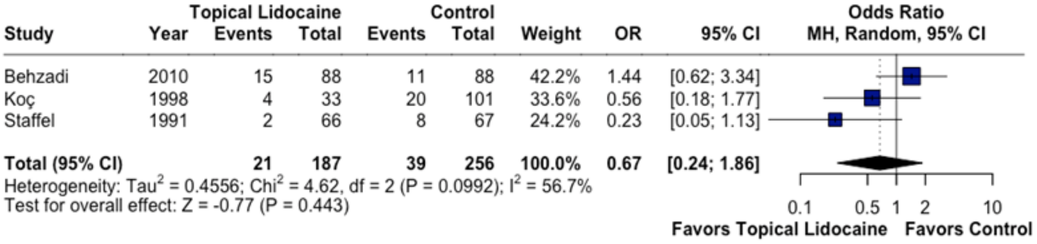

E.

Study	Year	Topical Lidocaine Events	Topical Lidocaine Total	Control Events	Control Total	Weight	OR	95% CI
Behzadi	2010	34	88	31	88	63.1%	1.16	[0.63; 2.14]
Oliveira	2024	3	40	2	40	9.6%	1.54	[0.24; 9.75]
Soares	2016	6	82	11	82	27.2%	0.51	[0.18; 1.45]
**Total (95% CI)**		**43**	**210**	**44**	**210**	**100.0%**	**0.95**	**[0.53; 1.71]**

Heterogeneity: $Tau^2 = 0.0442$; $Chi^2 = 2.02$, df = 2 (P = 0.3649); $I^2 = 0.8\%$
Test for overall effect: Z = -0.17 (P = 0.869)

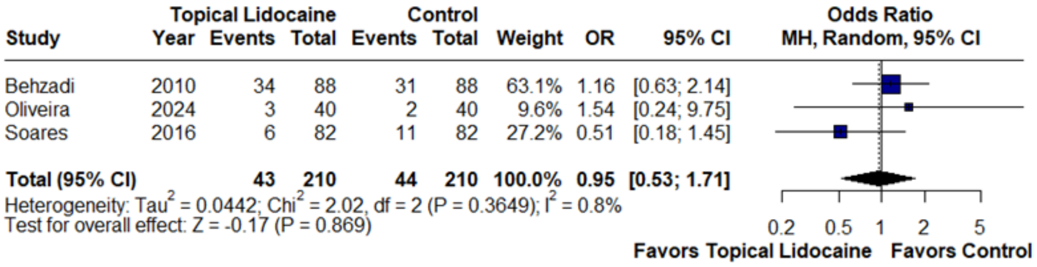

Supplementary Figure 7. Risk of bias in randomised trials with the RoB-2 tool, illustrated in a summary plot (A) and a traffic light plot (B).

A.

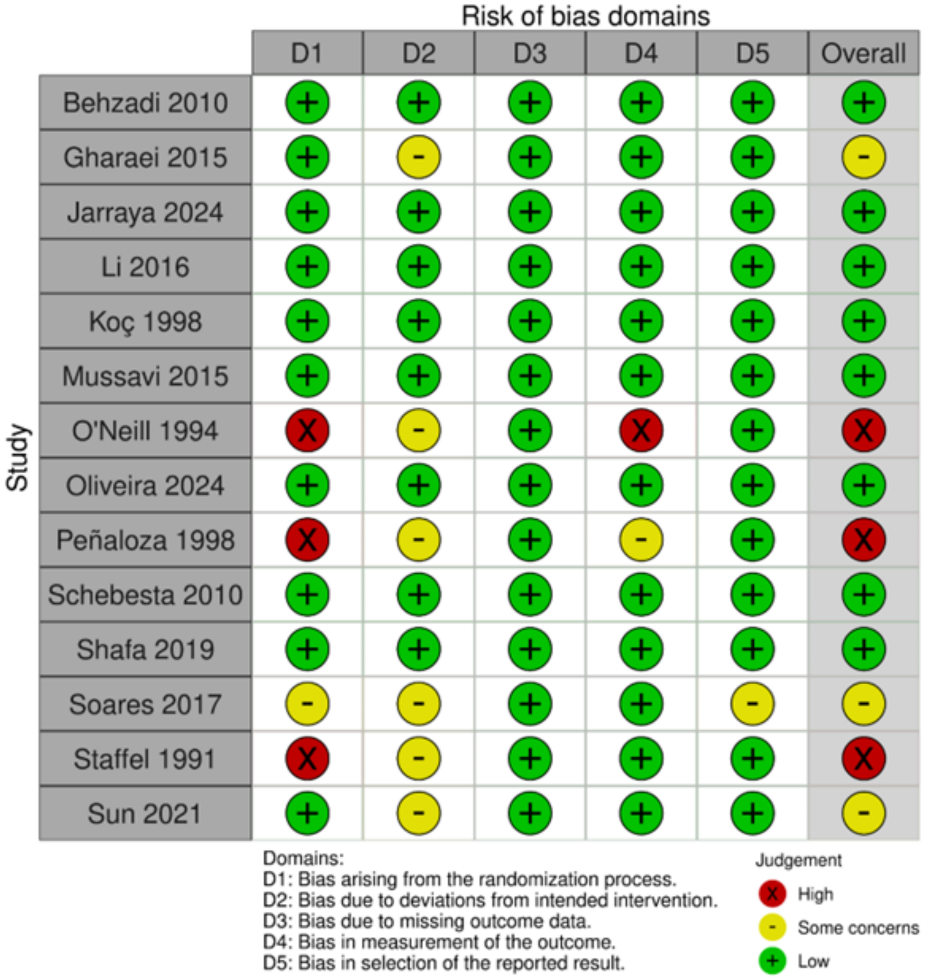

B.

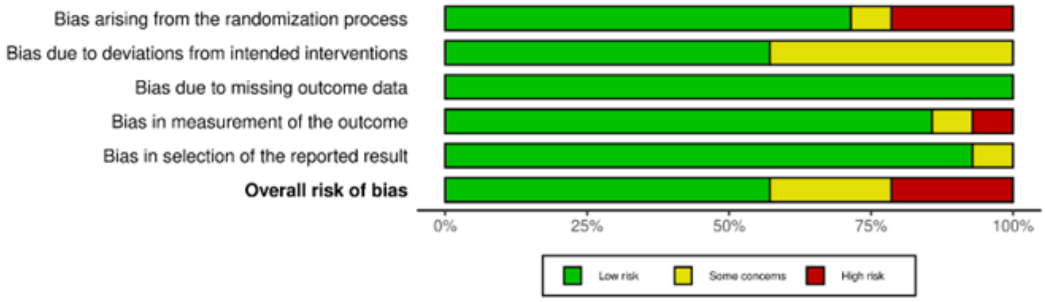

Supplementary Figure 8. Risk of bias in non-randomised cohorts with the ROBINS-I tool, illustrated in a summary plot (A) and a traffic light plot (B).

A.

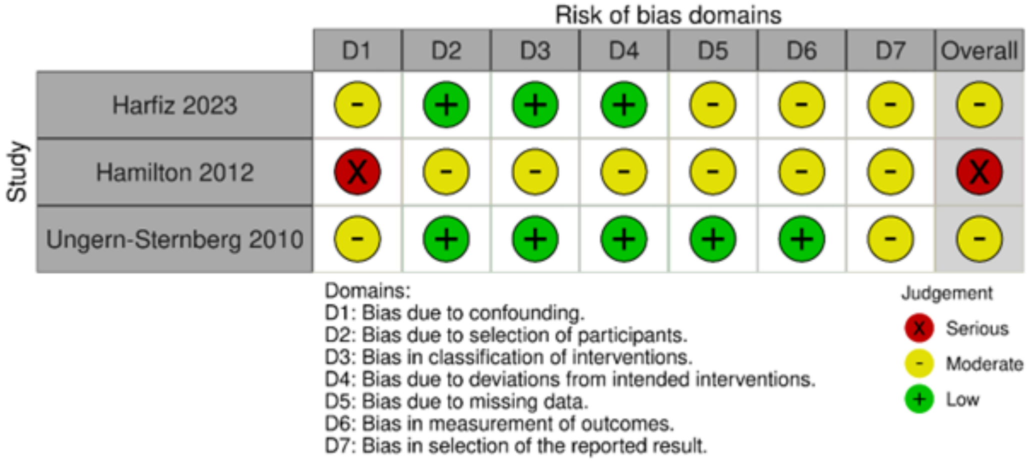

B.

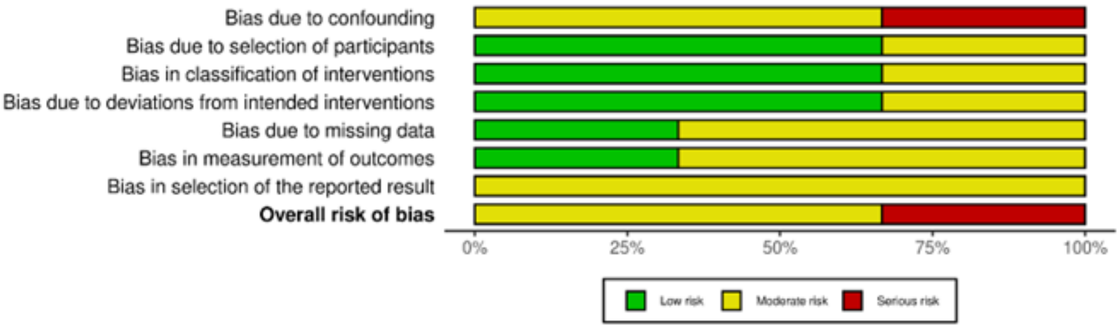

Supplementary Figure 9. Leave-one-out sensitivity analyses for the primary outcomes of laryngospasm (A), cough (B) and desaturation (C).

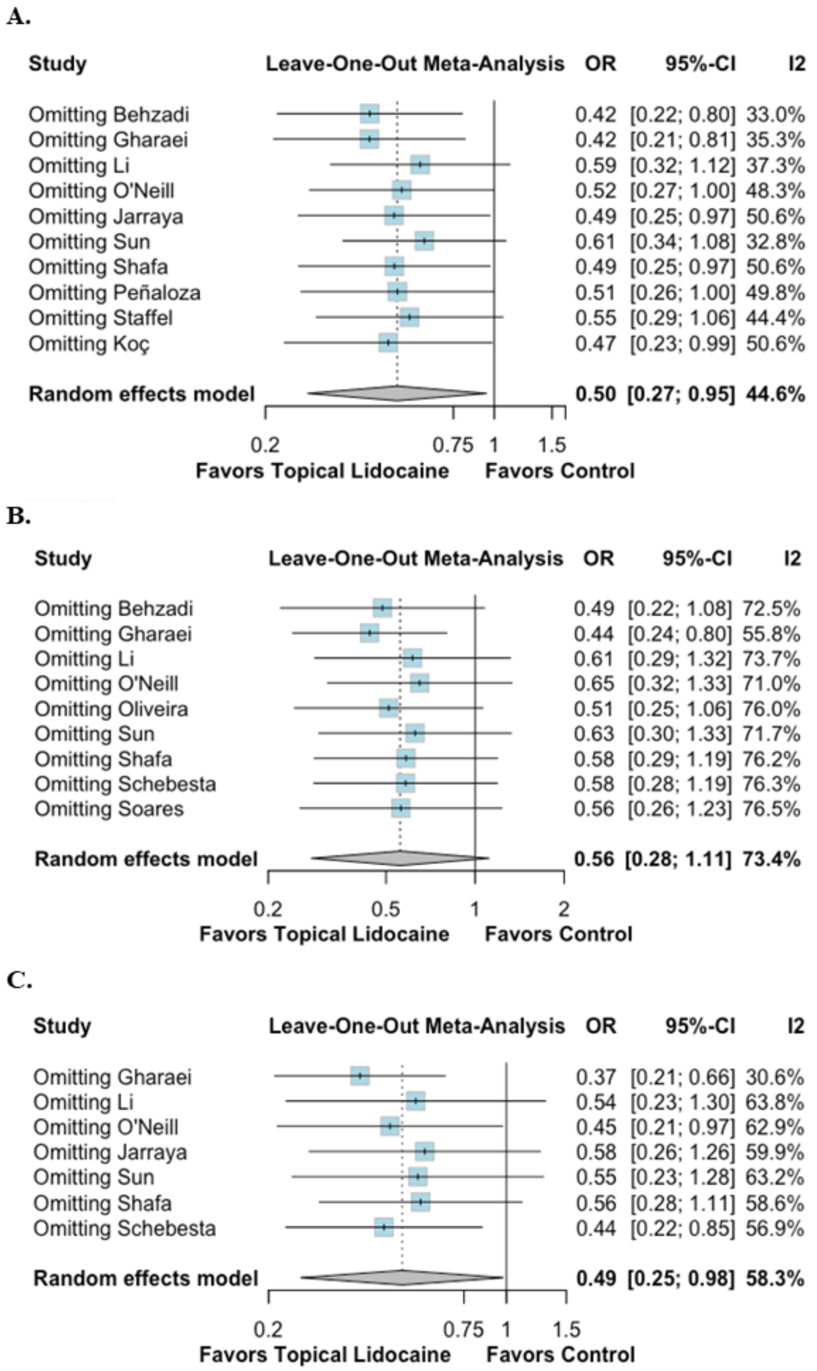

Supplementary Figure 10. Meta-regression sensitivity analyses of laryngospasm with potential moderators, including age (A), sample size (B), control groups (C), airway management devices (D), and type of lidocaine administration (E).

A.

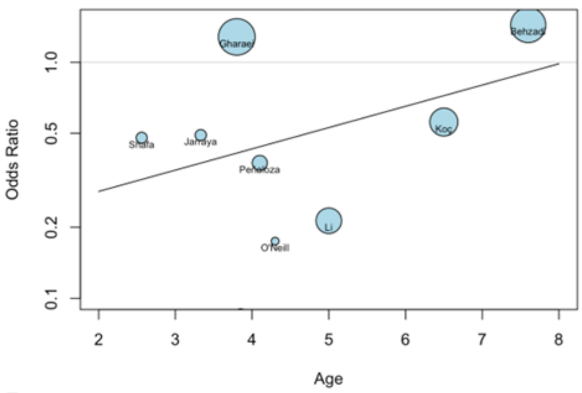
Odds Ratio
1.0
0.5
0.2
0.1
Gharaei
Behzadi
Shafa
Jarraya
Koc
Penaloza
Li
O'Neill
2
3
4
5
6
7
8
Age

B.

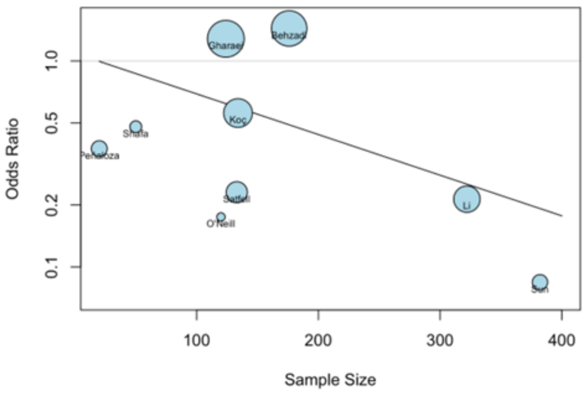
Odds Ratio
1.0
0.5
0.2
0.1
Gharaei
Behzadi
Koc
Shafa
Penaloza
Saffell
O'Neill
Li
Sun
100
200
300
400
Sample Size

C.

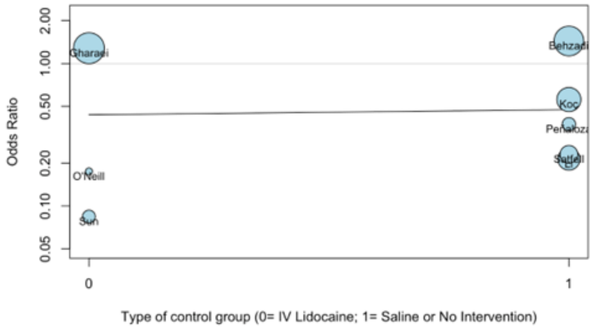
Odds Ratio
2.00
1.00
0.50
0.20
0.10
0.05
Gharaei
Behzadi
Koc
Penaloza
Saffell
O'Neill
Sun
0
1
Type of control group (0= IV Lidocaine; 1= Saline or No Intervention)

D.

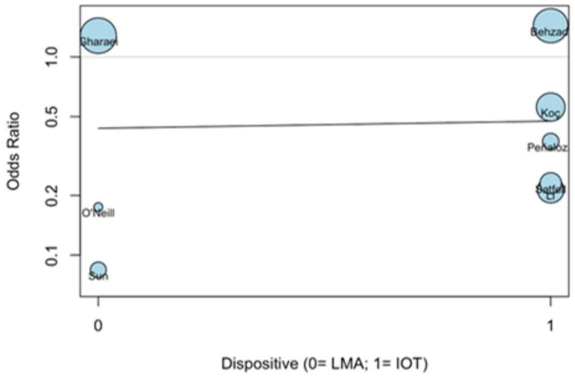
Odds Ratio
1.0
0.5
0.2
0.1
Gharaei
Behzadi
Koc
Penaloz
Saffell
O'Neill
Sun
0
1
Dispositive (0= LMA; 1= IOT)

E.

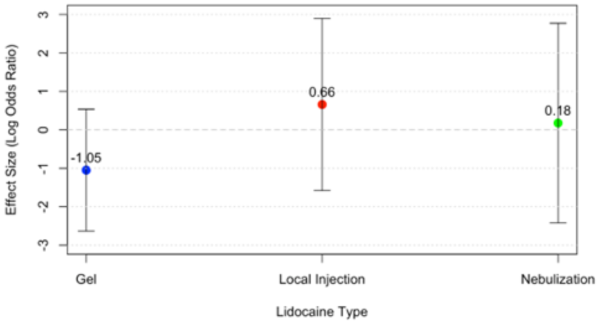
Effect Size (Log Odds Ratio)
3
2
1
0
-1
-2
-3
-1.05
0.66
0.18
Gel
Local Injection
Nebulization
Lidocaine Type

Supplementary Figure 11. Meta-regression sensitivity analyses of cough with potential moderators, including age (A), sample size (B), control groups (C), airway management devices (D), and type of lidocaine administration (E).

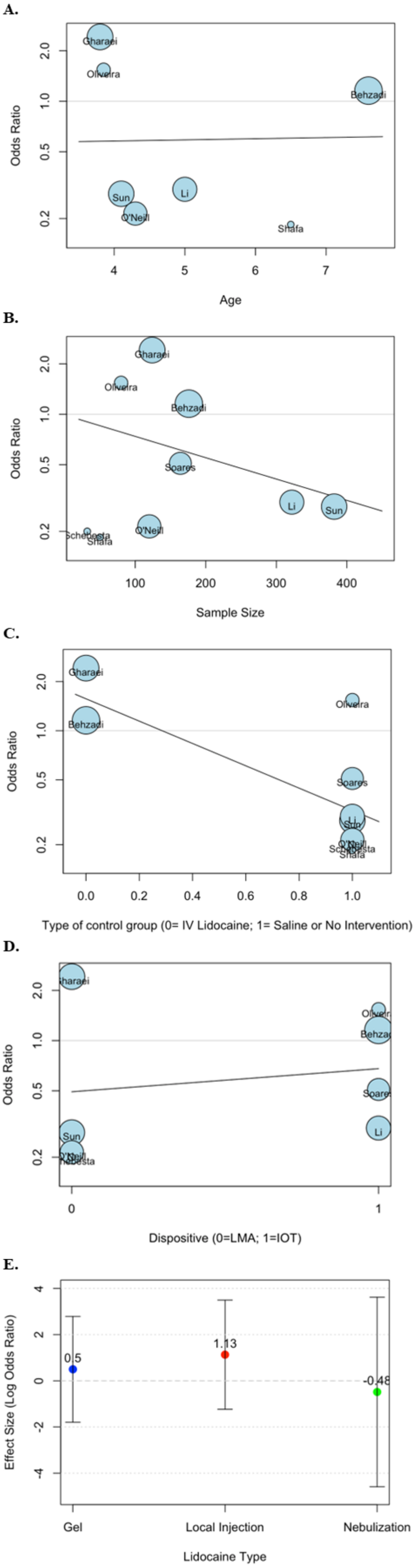
A.
Odds Ratio
Age
Gharaei
Olivera
Behzadi
Sun
Li
O'Neill
Shafa
B.
Odds Ratio
Sample Size
Soares
Schenesta
C.
Odds Ratio
Type of control group (0= IV Lidocaine; 1= Saline or No Intervention)
D.
Odds Ratio
Dispositive (0=LMA; 1=IOT)
E.
Effect Size (Log Odds Ratio)
0.5
1.13
-0.48
Gel
Local Injection
Nebulization
Lidocaine Type

Supplementary Table 1. Baseline characteristics of observational studies included

Study, year	Design	Patients, n<br>TAL / CTRL	Male[†], %<br>TAL / CTRL	Age[†], years<br>TAL / CTRL	Weight, kg<br>TAL / CTRL	TAL concentration and formulation	Control description	Airway Management	Surgery/Procedure characteristics	ASA classification, n<br>TAL / CTRL
Hafiz,[39] 2023	OP	60 / 30	NR	2% TAL group: 7.2 ± 4.36<br>10% TAL group: 7.1 ± 4.37 /<br>CTRL: 6.3 ± 4.09	2% TAL group: 27.8 ± 11.4<br>10% TAL group: 27.3 ± 11 /<br>CTRL: 26.6 ± 12.4	2% and 10% Lidocaine spray	IV lidocaine	EET	NR	ASA I and ASA II
Hamilton,[38] 2012	OP	TAL onto vocal cords: 254<br>TAL into pharynx: 236 /<br>CTRL: 510	TAL onto vocal cords: 52<br>TAL into pharynx: 50.4 /<br>CTRL: 51.5	TAL onto vocal cords: 5 [0–15]<br>TAL into pharynx: 5 [0–16] /<br>CTRL: 5 [0–16]	TAL onto vocal cords: 22.8 [2.5–72.5]<br>TAL into pharynx: 25.2 [2.8–93.5] /<br>CTRL: 26.4 [2.5–88.9]	4% Lidocaine spray	No TAL	ETI without NMB	ENT surgery, plastic surgery, orthopaedics, dental surgery, and general surgery	NR
von Ungern-Sternberg,[37] 2010[‡]	OP	558 / 2234	59.7	6.21 ± 4.8.	NR	NR% Lidocaine spray	No TAL	ETI	Surgical or medical procedures under general anaesthesia	ASA I: 4565<br>ASA II: 3394<br>ASA III: 1202<br>ASA IV: 4 123

[†]: Mean ± standard deviation or mean (range) or median [range]; [‡]: Overall trial baseline characteristics were based on the total study population; ASA: American Society of Anesthesiologists; CTRL: control; EET: endotracheal extubation; ENT: Ear, Nose, and Throat surgery; ETI: endotracheal intubation; ETT: endotracheal tube; IV: intravenous; NR: not reported; OP: observational prospective study; TAL: topical lidocaine.

Supplementary Table 2. Summary of Subgroup Analyses Findings.

Subgroup Analysis	Outcomes		Odds ratio (95% CI)	I² (%)	No. of studies	Intervention	Control
						No. of events/total	No. of events/total
**Lidocaine formulation sub-analysis**	*Gel / Cream*	Laryngospasm	0.33 (0.04-2.70)	73.4	3	20 / 316	30 / 310
		Cough	0.47 (0.12-1.92)	86.3	4	44 / 328	69 / 330
		Desaturation	0.90 (0.33-2.45)	60	4	25 / 328	35 / 330
	Nebulisation	Laryngospasm	0.43 (0.12-1.61)	0	3	4 / 95	8 / 95
	Local injection	Cough	0.97 (0.58-1.63)	0.8	3	43 / 210	44 / 210
**Airway management sub-analysis**	*Endotracheal intubation / extubation*	Laryngospasm	0.49 (0.21-1.14)	52.1	5	26 / 359	56 / 426
		Cough	0.67 (0.31-1.44)	58.2	4	50 / 372	65 / 370
	Laryngeal mask airway	Laryngospasm	0.33 (0.04-2.70)	73.46	3	20 / 316	30 / 310
		Cough	0.47 (0.12-1.92)	86.3	4	44 / 328	69 / 330
		Desaturation	0.90 (0.33-2.45)	60	4	25 / 328	35 / 330
**Lidocaine concentration sub-analysis**	*1% lidocaine*	Cough	0.64 (0.23-1.78)	0	3	6 / 106	15 / 147
	2% lidocaine	Laryngospasm	0.71 (0.36-1.41)	44	6	42 / 466	65 / 530
		Cough	0.61 (0.23-1.68)	82.4	5	76 / 385	93 / 389
		Desaturation	0.66 (0.28-1.57)	64	5	34 / 357	59 / 361
**Children's age sub-analysis**	*Preschoolers*	Laryngospasm	0.34 (0.10-1.10)	63.3	5	25 / 488	47 / 480
		Cough	0.43 (0.15-1.28)	83.1	5	51 / 490	90 / 490
		Desaturation	0.56 (0.28-1.11)	58.6	6	42 / 550	81 / 550
	School-aged	Laryngospasm	0.67 (0.24-1.86)	56.7	3	21 / 187	39 / 256

		Cough	0.95 (0.53-1.71)	0.8	3	43 / 210	44 / 210

Supplementary Table 3. GRADE Approach to Ascertain Certainty of Evidence.

Certainty assessment							№ of patients		Effect		Certainty	Importance
**№ of studies**	**Study design**	**Risk of bias**	**Inconsistency**	**Indirectness**	**Imprecision**	**Other considerations**	**Topical lidocaine**	**Control Group**	**Relative (95% CI)**	**Absolute (95% CI)**		
**Laryngospasm**												
10	randomised trials	serious	not serious	not serious	not serious	none	48/760 (6.3%)	90/821 (11.0%)	**OR 0.50** (0.27 to 0.95)	**52 fewer per 1.000** (from 77 fewer to 5 fewer)	⨁⨁⨁◯ Moderate	IMPORTANT
**Cough**												
9	randomised trials	not serious	serious	not serious	not serious	none	94/725 (13.0%)	136/725 (18.8%)	**OR 0.56** (0.28 to 1.11)	**73 fewer per 1.000** (from 127 fewer to 16 more)	⨁⨁⨁◯ Moderate	IMPORTANT
**Desaturation**												
7	randomised trials	not serious	not serious	not serious	not serious	none	43/575 (7.5%)	89/575 (15.5%)	**OR 0.49** (0.25 to 0.98)	**72 fewer per 1.000** (from 111 fewer to 3 fewer)	⨁⨁⨁⨁ High	IMPORTANT

CI: confidence interval; **OR:** odds ratio
